# A genomic atlas of human adrenal and gonad development

**DOI:** 10.12688/wellcomeopenres.11253.2

**Published:** 2017-10-23

**Authors:** Ignacio del Valle, Federica Buonocore, Andrew J. Duncan, Lin Lin, Martino Barenco, Rahul Parnaik, Sonia Shah, Mike Hubank, Dianne Gerrelli, John C. Achermann

**Affiliations:** 1 Genetics and Genomic Medicine, UCL Great Ormond Street Institute of Child Health, London, UK; 2 Developmental Biology and Cancer, UCL Great Ormond Street Institute of Child Health, London, UK; 3 Institute for Molecular Bioscience, University of Queensland, Brisbane, Australia; 4 Institute of Cardiovascular Science, University College London, London, UK; 5 The Centre for Molecular Pathology, Royal Marsden Hospital, Sutton, UK

**Keywords:** adrenal, testis, ovary, sex development, steroidogenesis, germ cell, human development, gene expression

## Abstract

**Background**: In humans, the adrenal glands and gonads undergo distinct biological events between 6-10 weeks post conception (wpc), such as testis determination, the onset of steroidogenesis and primordial germ cell development. However, relatively little is currently known about the genetic mechanisms underlying these processes. We therefore aimed to generate a detailed genomic atlas of adrenal and gonad development across these critical stages of human embryonic and fetal development.

**Methods**: RNA was extracted from 53 tissue samples between 6-10 wpc (adrenal, testis, ovary and control). Affymetrix array analysis was performed and differential gene expression was analysed using Bioconductor. A mathematical model was constructed to investigate time-series changes across the dataset. Pathway analysis was performed using ClueGo and cellular localisation of novel factors confirmed using immunohistochemistry.

**Results**: Using this approach, we have identified novel components of adrenal development (e.g. *ASB4*, *NPR3*) and confirmed the role of *SRY *as the main human testis-determining gene. By mathematical modelling time-series data we have found new genes up-regulated with *SOX9* in the testis (e.g. *CITED1*), which may represent components of the testis development pathway. We have shown that testicular steroidogenesis has a distinct onset at around 8 wpc and identified potential novel components in adrenal and testicular steroidogenesis (e.g. *MGARP*, *FOXO4*, *MAP3K15*, *GRAMD1B*, *RMND2*), as well as testis biomarkers (e.g. *SCUBE1*). We have also shown that the developing human ovary expresses distinct subsets of genes (e.g. *OR10G9*, *OR4D5*), but enrichment for established biological pathways is limited.

**Conclusion**: This genomic atlas is revealing important novel aspects of human development and new candidate genes for adrenal and reproductive disorders.

## Introduction

The development of the adrenal gland and gonads (testes, ovaries) are two of the most important embryological events, but relatively little is known about the exact mechanisms of these processes in humans.

**Figure 1.**
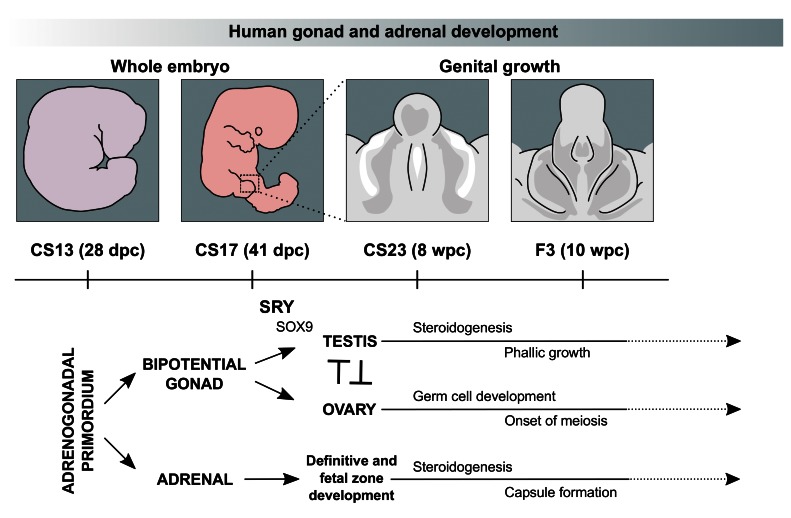
Current model of human gonad and adrenal development between Carnegie Stage 13 (CS13) and Fetal Stage 3 (F3). The bipotential gonad and adrenal gland both arise from the adrenogonadal primordium around CS13. Sex determination in the bipotential gonad occurs between CS17 and CS23 when transient *SRY* expression promotes the upregulation of genes, including *SOX9*, in the developing testis. Sex differentiation starts with the onset of steroidogenesis around CS23, which results in development of the external genitalia and phallic growth (right panel). Ovary-specific genes are thought to suppress male pathway specification. The ovary also supports germ cell expansion and entry into meiosis. The fetal adrenal cortex develops into definitive and fetal zones, producing steroid hormones and adrenal androgens. Chromaffin cells derived from the sympathetic nervous system migrate into the developing adrenal gland, merging later to form the adrenal medulla.

Development of these structures is closely linked as they arise from a shared region of intermediate mesoderm in early embryogenesis and subsequently have common biological processes, such as the ability to synthesise steroid hormones (see Figure 1 for an overview).

The primordial adrenal gland arises as a distinct structure at around 28 days post-conception (dpc) and undergoes rapid growth during late embryonic and early fetal life ( Ishimoto & Jaffe, 2011). The adrenal cortex develops into definitive and fetal zones that can produce steroid hormones (cortisol, aldosterone) and large amounts of adrenal androgens, such as dehydroepiandrosterone (DHEA) ( Miller & Auchus, 2011; Xing *et al.*, 2015). Chromaffin cells derived from the sympathetic nervous system migrate into the developing adrenal gland and later coalesce to form the adrenal medulla, which secretes adrenaline and noradrenaline ( Figure 1). It has been proposed that the entire hypothalamic-pituitary-adrenal (HPA) axis is functionally intact during early fetal life ( Goto *et al.*, 2006).

In contrast, the developing gonad remains “bipotential” until around 41 dpc as it can differentiate into either a testis or ovary ( Figure 1) ( Bashamboo *et al.*, 2017; Svingen & Koopman, 2013).

In the developing 46,XY embryo, transient expression of the testis-determining gene, *SRY*, on the Y chromosome is believed to lead to upregulation of genes such as *SOX9* and the process of testis determination ( Bashamboo *et al.*, 2017; Hanley *et al.*, 2000; Hiramatsu *et al.*, 2009; Koopman *et al.*, 2016; Sekido & Lovell-Badge, 2008). At around 7–8 weeks post conception (wpc), Sertoli cells secrete the peptide hormone Anti-Müllerian Hormone (AMH, also known as Müllerian Inhibiting Substance, MIS) to regress Müllerian structures (uterus and upper vagina). Fetal Leydig cells develop and synthesise testosterone. Testosterone stabilises Wolffian structures and stimulates development of the external genitalia through its peripheral conversion to dihydrotestosterone ( Figure 1) ( Miller & Auchus, 2011).

Ovary development in the 46,XX embryo was originally thought to be a “passive” process that occurs in the absence of SRY and other components of the testis-determining pathway. Although the morphological changes in the ovary are less striking, data from mice and from 46,XX individuals with rare forms of ovotestis development suggest that distinct pathways are up-regulated (e.g. RSPO1/WNT4/β-catenin), which may function largely to repress testis development ( Figure 1) ( Bashamboo *et al.*, 2017; Beverdam & Koopman, 2006; Bouma *et al.*, 2007; Jameson *et al.*, 2012; Nef *et al.*, 2005). The ovary also has expansion of primordial germ cells starting towards the end of this early fetal period ( Fowler *et al.*, 2009).

Given the limited data currently available ( Gerrard *et al.*, 2016; Houmard *et al.*, 2009; O’Shaughnessy *et al.*, 2007; Ostrer *et al.*, 2007), we aimed to generate a genomic atlas of human adrenal and gonad development between 6 and 10 wpc. This period represents a critical window for organogenesis during which many key biological events occur in tissue specification and cellular differentiation. This unique dataset is supported by our current knowledge of key genetic components in these processes and by human conditions affecting adrenal and gonadal function. By comparing datasets at different times and in different tissues, as well as by mathematical modelling of time series data, several novel insights into human adrenal and gonad development are emerging.

## Methods

### Tissue samples

Human embryonic and fetal tissue samples used in this study were obtained in collaboration with the MRC/Wellcome Trust-funded Human Developmental Biology Resource ( HDBR). The HDBR is a tissue bank regulated by the Human Tissue Authority. Samples were collected with appropriate maternal written consent and with approval from the NRES Committee London-Fulham (REC reference 08/H0712/34+5). Material was obtained only from surgical terminations of pregnancy to avoid any potentially interfering effects of anti-progestogens on nuclear receptor gene transcription.

All materials were kept on ice and processed rapidly after collection. The adrenal glands, gonads and any control tissues needed were identified and removed by blunt dissection under a dissecting microscope. Samples were immediately immersed in RNAlater® (Ambion, Austin, TX, USA) and stored at -20 C. The age of the embryonic or fetal sample was calculated based on morphological characteristics using published guidelines ( Hern, 1984; O’Rahilly & Müller, 1987) by a single experienced researcher (DG) ( Supplementary Table 1). Each tissue sample was also karyotyped using standard G-banding, to determine the sex of the embryo/fetus and also to ensure there were no significant aneuploidies or chromosomal rearrangements present.

A total of 53 different organs between 42 days post conception (dpc) (Carnegie Stage 17) and 10 weeks post conception (wpc) (Fetal Stage 3) were included in the analysis. This dataset consisted of 17 adrenal glands, 20 testes and 10 ovaries, as well as 6 “control” samples (46, XY) from spine, brain, muscle, heart, kidney and liver. The adrenal gland and testis samples came from the same fetus in 11 cases across the age range. Control samples were obtained from 2 of these. An overview of all samples is provided in Supplementary Table 1.

### RNA extraction

RNA was extracted using the TRIzol method. In brief, the tissue sample was removed from RNAlater® and homogenized in 1ml TRIzol reagent using a Pellet Pestle micro-grinder (Kontes/Kimble Chase, USA). Chloroform (0.2 ml) was added to allow phase separation, and RNA was precipitated from aqueous phase by addition of 0.5 ml isopropyl alcohol. The RNA pellet was washed in 1ml ice-cold 75% ethanol and air dried before resuspension in nuclease-free water. The concentration and A260/A280 ratio was measured using a NanoDrop ND-1000 spectrophotometer (NanoDrop Technologies, Witec, Littau, Switzerland). RNA was stored at -80 C prior to use.

### Arrays

Expression assay was performed using the Affymetrix GeneChip Human Gene 1.0 ST array, covering approximately 36000 RefSeq transcripts. In brief, RNA concentration and quality was assessed using an Agilent Bioanalyzer. 250ng of total RNA was converted to ssDNA using the Ambion Whole Transcript Sense Target Labeling Assay and labelled and prepared for hybridisation using the Affymetrix Genechip WT Terminal Labelling and Hybridisation kits, as per the manufacturer’s protocol. Hybridisation was performed for 16 hours at 45 C. Arrays were washed and stained using the Affymetrix Fluidics Station 450 and scanned on the Affymetrix Genechip 3000S scanner as per the manufacturer’s instructions.

After passing quality control, CEL files were normalised using the Robust Multi-array Average (RMA) algorithm (oligo package in R, version 1.38.0). Adjustments for batch effects was performed using ComBat (sva package, version 3.22.0) ( Leek *et al.*, 2012). One adrenal sample that was an extreme outlier in several QC platforms was excluded prior to further analysis (leaving the 17 adrenal samples reported above). Statistical analysis of the microarray data was carried out in R programming environment (version 3.3.3 running under Ubuntu 16.04.2) using Bioconductor packages ‘oligo’( Carvalho & Irizarry, 2010) and ‘limma’ (version 3.30.12) ( Smyth, 2004). The hierarchical cluster dendrogram was generated using the agglomeration method ward.D2. Differentially expressed genes were obtained using linear models and the Benjamini and Hochberg method was applied to adjust the P-values for multiple testing ( Benjamini & Hochberg, 1995). The cut-off values for adjusted P-values and log _2_ fold-changes were 0.05 and 1 or 2, respectively. The pheatmap (version 1.0.8) and VennDiagram packages (version 1.6.17) were used to generate the heatmaps and Venn diagrams, respectively. Pathway enrichment analysis was carried out using the Cytoscape plug-in ClueGO (version numbers 3.4.0 and 2.3.3, respectively) ( Bindea *et al.*, 2009).

Array data are available from the ArrayExpress database under the accession number E-MTAB-5525.

### A model of expression changes over time

A phenomenological model (“BALT model”) was developed to pinpoint the time when gene expression levels change, as well as evaluating the speed and magnitude of this transition. The general form of the model is a sigmoid function relating the transcript concentration *X* (on a 2-log scale) to the time elapsed since conception ( *t*, expressed in days):

$$X(t)=B+\frac{A}{1\;+\;e^{f(T)*(L–t)}}$$

In the formula above, B (“basis”) is the basic expression level at the start of the time course and A (“amplitude”) is the amplitude of the difference between the finishing expression level and the starting one. For down-regulated genes this latter parameter takes negative values. The parameter L (“localisation”) captures the inflexion point of the sigmoid, and hence can identify at which point in time a gene undergoes a change of expression level. Finally, T (“Transition”) is a measure of the speed of this transition and the sub-function f was chosen so that this parameter can also be expressed in days.

The model was fitted to the data for each gene using a least square objective function (maximum likelihood) with a gradient following algorithm (Levenberg-Marquardt). With this, the values for the aforementioned parameters that best fit the data were obtained as well as an evaluation of the goodness of fit (and thus, of the validity of the model). Furthermore, robustness of the parameters were estimated using a Markov Chain Monte Carlo procedure.

It was found that most of the differentially expressed genes in our biological system could be well described by this model or simplified versions of it. Hence the procedure was seen as a way to compress high dimensional data into a smaller set of easily interpretable indicators. This modelling framework allowed genes to be grouped according to their transition times between different expression levels in a developmental process as well as the manner of this transition. This model was used predominantly to investigate time course data in the testis. The two main groups of genes that were analysed were those that followed a curvilinear upregulation in expression from the start of the dataset similar to SOX9, as well as genes that showed a sigmoidal upregulation in expression with the onset of steroidogenesis. The mid-point (in dpc) of the Carnegie or Fetal Stage was used when analysing data for the model ( Supplementary Table 1).

### Quantitative RT-PCR

Purified RNA from adrenal glands or testis and control tissue (liver, brain, muscle) was quantified using the NanoDrop 1000 spectrophotometer (Thermo Fisher Scientific). First-strand cDNA was synthesised using SuperScript II Reverse Transcriptase (Invitrogen) and random primers according to the manufacturer’s instructions. Taqman® probes were obtained for the following genes of interest: *MAP3K15*, *ASB4*, *TDGF1*, *FOXO4*, *NRK*, *CITED1, ZNF280B, PRPS2, ANKRD18A, GRAMD1B* and *RMDN2* (Applied Biosystems, Warrington, UK). Amplification was performed in a total volume of 20 µl per reaction using TaqMan® Gene Expression Master Mix on the StepOnePlus™ System (Thermo Fisher Scientific). Three biological replicates were used for each group. The relative quantification of gene expression was calculated as 2 ^-ΔΔCt^ using the comparative Ct (ΔΔCt) method and GAPDH as the housekeeping internal control. Data were analysed with StepOne software (v 2.1) and results expressed as fold change above control. Standard error of the mean is shown.

### Immunohistochemistry

Human adrenal glands and testes of the relevant ages of interest were obtained with approval from the HDBR. Tissues were sunk and positioned in Tissue-Tek® O.C.T. (Fisher Scientific, Loughborough, UK) and frozen on dry ice. Frozen sections were cut at 12 µm thickness, collected on Superfrost slides (Fisher Scientific), dried and stored at -20 C. For immunohistochemistry, sections were fixed briefly in 4% PFA in TBS, rinsed in TBS and blocked for 1 hour in 1% BSA in TBS-Tween (0.5% Tween), before being incubated overnight with the following primary antibodies: NR5A1 (Invitrogen N1665/434200, 1:200), CYP11A1 (Sigma HPA016436, 1:20), NRK (Sigma HPA017238, 1:250), SOX9 (Sigma HPA001758, 1:200), CITED1 (Abcam ab15036, 1:200), GRAMD1B (Sigma HPA008557, 1:200; Abnova PAB15705, 1:200), RMDN2 (Sigma HPA034706, 1:200), FOXO4 (Cell Signalling 2499, 1:200), AMH (Abcam ab103233 1:200), INSL3 (Sigma HPA028615, 1:1000), SCUBE1 (Abcam ab105358, 1:50). The next morning, sections were washed in TBS-Tween and incubated for 1 hour with the relevant secondary antibodies: Alexa488 goat anti-mouse (Invitrogen A11001, 1:400) and Alexa555 goat anti-rabbit (Invitrogen A21429, 1:400). Nuclei were counterstained with DAPI (10 µg/ml, Sigma). Slides were washed with TBS-Tween and mounted using ProLong Gold Antifade Mountant (Life Technologies). Images were collected on a Zeiss LSM 710 confocal microscope (Carl Zeiss) and analysed using Zeiss Zen 2009 and Image J (version 1.46r).

## Results

### Generation of an atlas of gene expression in human adrenal gland, testis and ovary

Human embryonic and fetal tissues of interest were obtained following ethical approval and with informed consent from the Human Developmental Biology Resource (HDBR, www.hdbr.org). RNA was extracted and gene expression profiling performed using Affymetrix ST1.0 Gene arrays. A total of 53 different organs between 42 days (6 weeks) and 70 days (10 weeks) post-conception were included in the final analysis ( Supplementary Table 1). This dataset consisted of 17 adrenal glands (46,XY), 10 ovaries (46,XX), and 20 testes (46,XY), as well as 6 control samples (46,XY) from spine, brain, muscle, heart, kidney and liver. Adrenal and control samples with a 46,XY karyotype were intentionally chosen so that subtle effects of Y-chromosome genes on testis determination and sex development could be analysed. All relevant CEL files have been deposited in the ArrayExpress database (E-MTAB-5525) and the entire dataset of log _2_ normalised values is included in Dataset 1.

**Figure 2.**
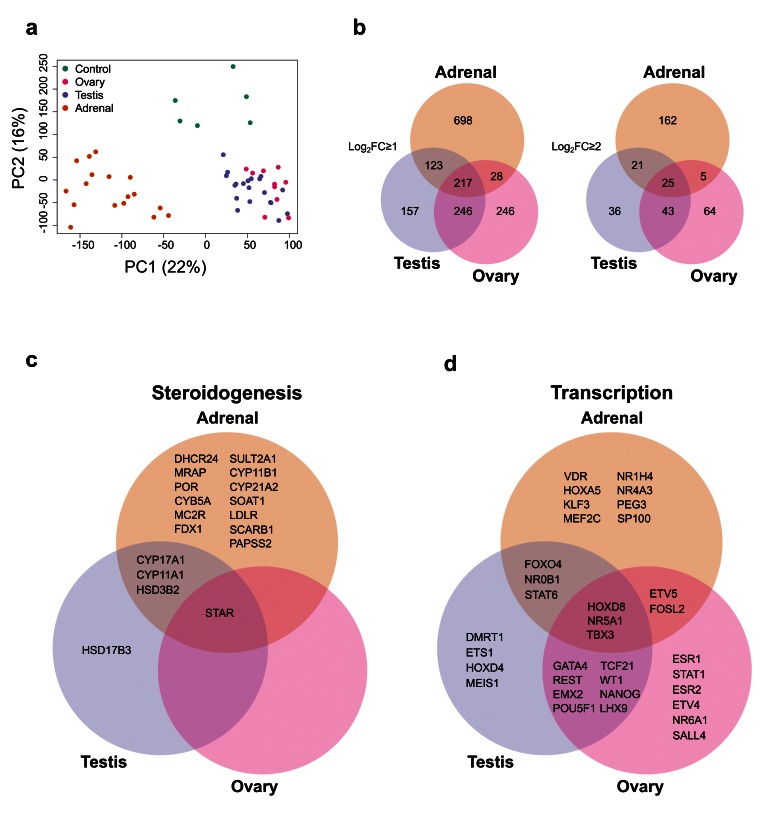
Differential gene expression in adrenal gland, testis and ovary compared to controls. ( **a**) Principal component analysis of the 53 samples included in the study (17 adrenal, 20 testes, 10 ovary and 6 controls). The scatter plot shows the position of samples based on the two first principal components (PC1, PC2). ( **b**) Venn diagram showing the overlap between differentially up-regulated genes in the adrenal, testis and ovary compared with control samples using either log _2_FC≥1 or log _2_FC≥2. ( **c**) Selected examples of differentially up-regulated genes (log _2_FC≥2) involved in steroidogenesis ( **d**) Selected transcription factors differentially expressed in each tissue (log _2_FC≥1). For all comparisons, *P*-value≤0.05.

**Figure 3.**
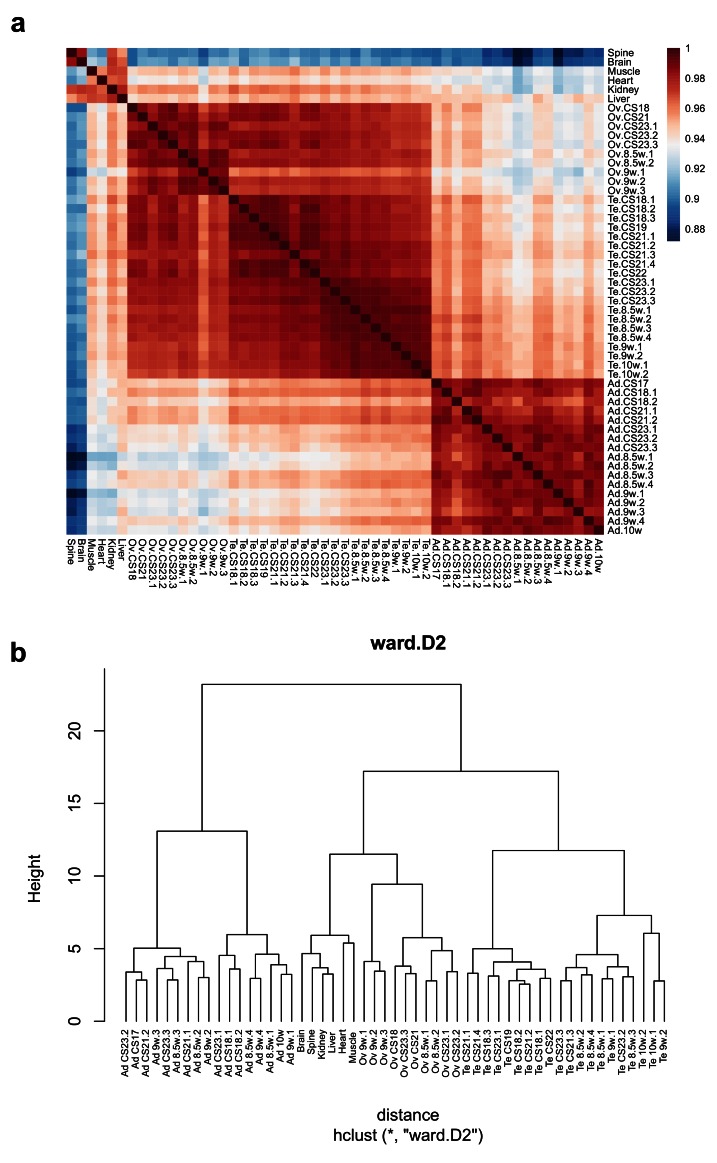
Cluster analysis for all 53 samples included in the study. ( **a**) Correlation plot (heatmap) of gene expression. ( **b**) Hierarchical cluster dendrogram using the agglomeration method ward.D2.

Initial comparisons of gene expression profiles in the different samples were undertaken using principal components analysis (PCA) and by generating correlation plots (heatmaps) and cluster dendrograms ( Figure 2a, Figure 3). Samples from each tissue clearly clustered together, with more similarity between testis and ovary profiles than adrenal gland ( Figure 2a, Figure 3).

### Global gene expression patterns reveal known disease-causing genes and identify novel tissue-specific factors

An analysis of global gene expression patterns was initially performed for the three tissues (adrenal, testis, ovary) compared to controls. Genes were identified that were up-regulated with a log _2_ fold change (FC) of ≥ 2 or ≥ 1 and adjusted *P*-value lower than 0.05 ( *P*≤ 0.05) ( Dataset 2– Dataset 4).

An overview of the number of differentially up-regulated genes that are specific to each tissue, or overlapping, is shown in Figure 2b. As expected, there was greater overlap between testis and adrenal (due to shared steroidogenic pathways) and testis and ovary (due to their gonadal origin and presence of germ cells) than between adrenal and ovary, which are developmentally and functionally more distinct.

Analysis of factors known to be involved in steroidogenesis showed that shared components of the steroidogenic pathways overlapped in the adrenal and testis datasets (e.g. *CYP11A1*, *HSD3B2*, *CYP17A1*), whereas the gene responsible for conversion of delta-4 steroids (e.g. androstenedione) into testosterone, *HSD17B3*, was the only testis-specific steroidogenic enzyme ( Figure 2c). Adrenal-specific steroidogenic components included the enzymes needed to make aldosterone and cortisol from precursors (e.g. *CYP11B1*, *CYP21A2*) as well as adrenal-specific receptors involved in signalling pathways (e.g. *MC2R* [also known as the ACTH receptor], *MRAP*).

An analysis of known transcription factors ( Figure 2d) revealed several key genes shared between the testis and ovary involved in gonad development (e.g., *WT1*, *EMX2*, *LHX9*, *GATA4*) or in germ cell maturation (e.g., *POU5F1* [also known as *OCT4*] and *NANOG*) ( Figure 2d). One key transcription factor shared between the adrenal and testis was the nuclear receptor *NR0B1* (also known as DAX-1), responsible for X-linked adrenal hypoplasia congenita with male infertility ( Suntharalingham *et al.*, 2015). Another key nuclear receptor common to all three tissues was *NR5A1* (also known as steroidogenic factor-1), responsible for adrenal insufficiency, testicular dysgenesis and primary ovarian insufficiency ( Achermann *et al.*, 1999; Lourenço *et al.*, 2009; Suntharalingham *et al.*, 2015). Therefore, the dataset seemed validated based on known factors involved in development as well as in genetic conditions in humans.

### Known and novel adrenal-specific genes

**Figure 4.**
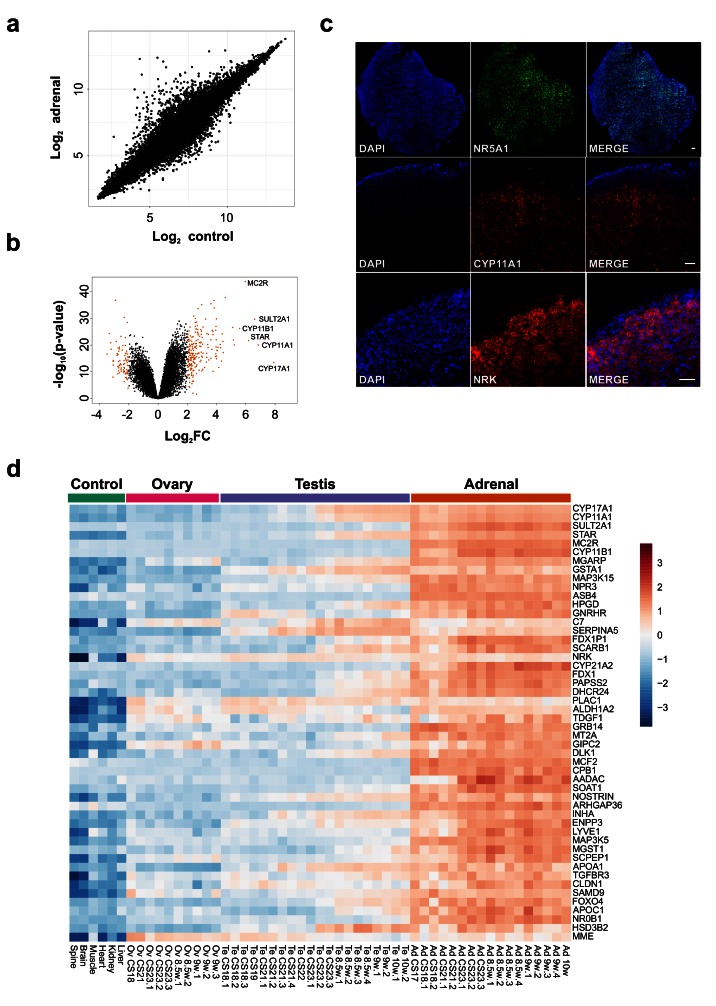
Differential expression of genes in the adrenal gland. ( **a**) Correlation plot for log _2 _mean gene expression levels in the adrenal versus control samples (Pearson’s product-moment correlation test correlation coefficient=0.96, *P*-value≤ 2.2e-16). ( **b**) Volcano plot analysis of differentially expressed genes in adrenal versus control samples. Genes with a log _2_FC≥2 (or log _2_FC≥-2) and *P*-value≤0.000005 are shown in orange. ( **c**) Immunohistochemistry of NR5A1, CYP11A1 and NRK in the human adrenal gland (9 wpc). Nuclei are stained blue with DAPI, which also highlights the outer capsule. Scale bars, 100 µm. ( **d**) Heatmap representing normalised gene expression values for the top 50 differentially up-regulated adrenal genes compared to control samples across the whole sample dataset. Genes are ordered according to descending log _2_FC values. The intensity of gene expression is indicated by a colour scale: blue for lowest and red for highest expression levels. For all samples shown, *P*-value≤1e-10.

**Figure 5.**
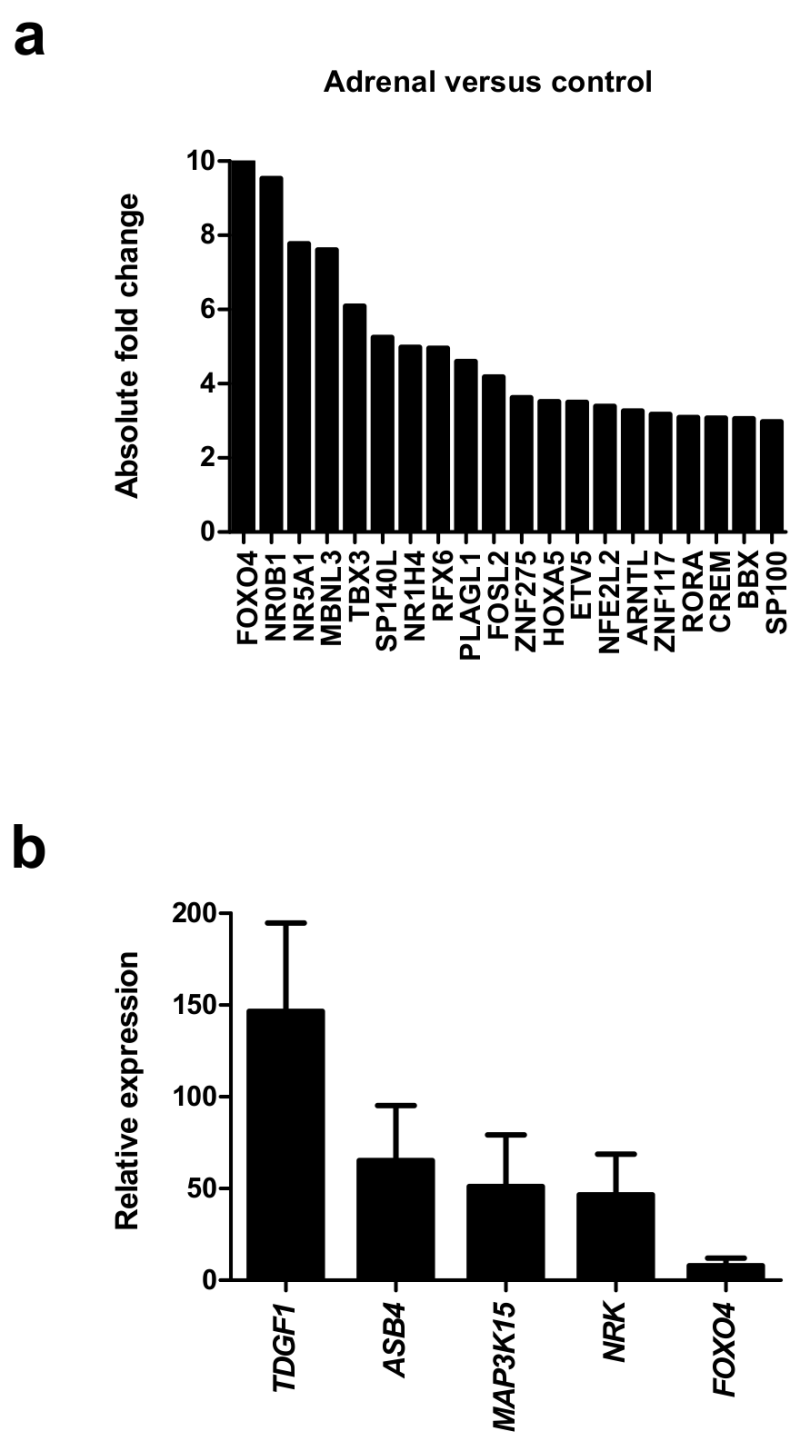
Analysis of differentially expressed adrenal genes. ( **a**) Absolute fold change (FC) of the top 20 up-regulated transcription factors in the adrenal gland compared to control samples. Data are derived from the array dataset. ( **b**) Confirmation of differential expression of several novel genes in the adrenal gland (9 wpc) by qRT-PCR. Pooled liver, brain and muscle were used as a control. *GAPDH* was used as a housekeeping gene.

Initial analysis focused on genes up-regulated in the adrenal gland compared to controls ( Figure 4a, Table 1 and Dataset 2). The most strongly differentially expressed adrenal genes were *CYP17A1* (absolute FC 240.2), *CYP11A1* (absolute FC 115.4), *SULT2A1* (absolute FC 97.3), *STAR* (absolute FC 73.4), *MC2R* (the ACTH receptor) (absolute FC 62.3) and *CYP11B1* (absolute FC 47.8) ( Figure 4a–d and Table 1). These genes are known core components of adrenal steroidogenesis and most are associated with monogenic disorders in humans ( Table 1). The most strongly differentially expressed transcription factors were *FOXO4* (absolute FC 10), *NR0B1* (DAX-1) (absolute FC 9.5) and *NR5A1* (SF-1) (absolute FC 7.8) ( Figure 5a). Key novel genes or those that have not been extensively studied in the adrenal gland before include *MGARP* (also known as *OSAP*), *MAP3K15*, *ASB4*, *NRK*, and *TDGF1* ( Figure 4d). Expression of these genes was confirmed by qRTPCR and/or immunohistochemistry ( Figure 4c, Figure 5b). Analysis of the heatmap for the top 50 differentially expressed genes revealed that several novel genes appear to be highly adrenal-specific (e.g., *ASB4*, *NPR3*, *GRB14*), whereas others are also expressed in the later testis samples, suggesting a potential novel role for these genes in steroidogenesis (see below) ( Figure 4d).

**Table 1.** The top 20 differentially expressed adrenal genes and other selected genes associated with monogenic disorders in humans. Genes are ranked based on log _2_FC in the adrenal (n=17) compared to control samples (n=6). Further clinical information is available at Online Mendelian Inheritance in Man ( OMIM).

Rank	Gene	log _2_FC	Fold change	OMIM gene	Clinical condition (monogenic)
1	CYP17A1	7.91	240.2	609300	Congenital adrenal hyperplasia (due to 17 α-hydroxylase deficiency)
2	CYP11A1	6.85	115.4	118485	Congenital adrenal hyperplasia (due to CYP11A1/ P450scc deficiency)
3	SULT2A1	6.60	97.3	-	
4	STAR	6.20	73.4	600617	Congenital lipoid adrenal hyperplasia; Familial glucocorticoid deficiency type 3
5	MC2R	5.96	62.3	607397	Familial glucocorticoid deficiency, type 1
6	CYP11B1	5.58	47.8	610613	Congenital adrenal hyperplasia (due to 11 β-hydroxylase deficiency)
7	MGARP	5.21	37.1	-	
8	GSTA1	5.15	35.5	-	
9	MAP3K15	5.11	34.5	-	
10	NPR3	4.62	24.6	-	
11	ASB4	4.60	24.3	-	
12	HPGD	4.46	22.0	601688	Cranioosteoarthropathy; Primary hypertrophic osteoarthropathy type 1
13	GNRHR	4.43	21.6	138850	Hypogonadotropic hypogonadism
14	C7	4.34	20.3	217070	C7 (complement) deficiency
15	SERPINA5	4.26	19.2	-	
16	FDX1P1	4.26	19.1	-	
17	SCARB1	4.15	17.8	601040	High-density lipoprotein elevation
18	NRK	4.09	17.1	-	
19	CYP21A2	4.09	17.1	613815	Congenital adrenal hyperplasia (due to 21-hydroxylase deficiency)
20	FDX1	4.03	16.3	-	
21	PAPSS2	4.00	16.0	603005	Brachyolmia type 4 with mild epiphyseal and metaphyseal changes, adrenal hyperandrogenism
22	DHCR24	4.00	15.9	606418	Desmosterolosis
25	TDGF1	3.96	15.6	187395	Forebrain defects
36	INHA	3.67	12.8	147380	Adrenocortical carcinoma (paediatric)
43	APOA1	3.42	10.7	107680	Apoplipoprotein A-I deficiency
46	SAMD9	3.34	10.2	610456	MIRAGE syndrome (includes growth restriction, adrenal hypoplasia, testicular dysfunction)
49	NR0B1 (DAX-1)	3.25	9.5	300473	X-linked adrenal hypoplasia congenita
50	HSD3B2	3.19	9.1	613890	Congenital adrenal hyperplasia (due to 3 β-hydroxysteroid dehydrogenase deficiency type 2)
60	NR5A1 (SF-1)	2.96	7.8	184757	DSD (testicular dysgenesis/impaired testosterone synthesis) +/- adrenal insufficiency
72	LDLR	2.81	7.0	600694	Familial hypercholesterolaemia
77	AMHR2	2.73	6.7	600956	Persistent Müllerian duct syndrome, type 2
105	POR	2.53	5.8	124015	P450 oxidoreductase deficiency/Antley-Bixler syndrome
117	MRAP	2.43	5.4	615410	Familial glucocorticoid deficiency, type 2
142	NPC1	2.31	5.0	607623	Niemann-Pick disease type C1

### SRY is the primary driver of human testis development

**Figure 6.**
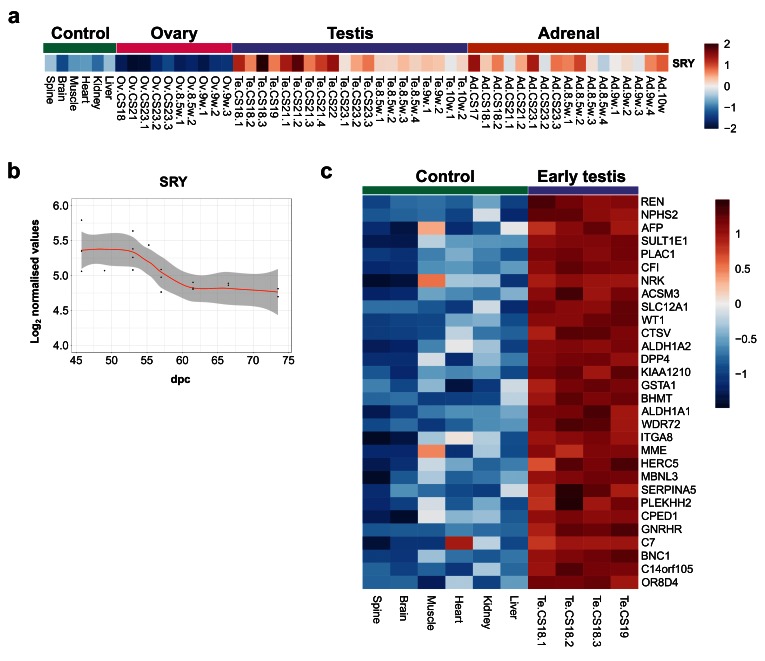
*SRY* expression in early human testis and differential expression of other genes. ( **a**) Heatmap showing normalised gene expression values for *SRY* across the whole sample dataset. ( **b**) Scatter plot of normalised gene expression of *SRY* in testis samples between 45 and 74 dpc. Loess method was used to fit a smooth curve between expression values. ( **c**) Heatmap representing normalised gene expression values for the top 30 differentially up-regulated genes in early testis samples (CS18 and CS19 stages) ( *N*=4) compared to control samples ( *N*=6). Genes are ordered according to decreasing log _2_FC values. The intensity of gene expression is indicated by a colour scale: blue for lowest and red for highest expression levels. For all samples shown, *P*-value≤0.05.

Studies in transgenic mice and humans with *SRY* deletions and translocations have suggested that *SRY* is the primary Y-chromosomal testis-determining gene in humans, but direct evidence is limited ( Bashamboo *et al.*, 2017; Hanley *et al.*, 2000; Koopman *et al.*, 2016). *SRY* is thought to be transiently up-regulated in the 46,XY bipotential gonad at around 42 dpc, leading to downstream expression of testis development pathways ( Hanley *et al.*, 2000). Analysis of *SRY* expression in early testis samples (CS18–CS19) compared to control tissues (all 46,XY) showed that *SRY* is the only significantly differentially-expressed protein coding Y-chromosomal gene (absolute FC 2, *P*-value≤0.003) ( Figure 6a and Dataset 5). Although changes are subtle, there was a clear transient expression of *SRY* that had occurred by the start of our dataset (around 45 dpc), with a reduction in intensity around CS23 (57 dpc) ( Figure 6a, Figure 6b).

**Figure 7.**
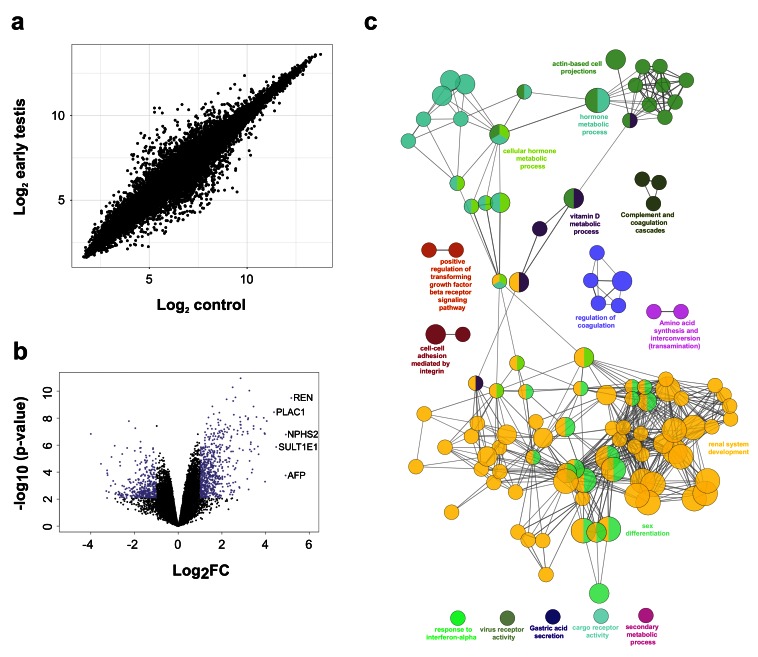
Differentially expressed genes in the early testis compared to control samples. ( **a**) Correlation plot for log _2 _mean gene expression levels in the early testis (CS18 to CS19) ( *N*=4) versus control samples ( *N*=6) (Pearson’s product-moment correlation test correlation coefficient=0.968, *P*-value≤ 2.2e-16). ( **b**) Volcano plot for the differentially expressed genes in early testis versus control samples. Purple colour indicates genes with absolute log _2_FC≥1 and *P*-value≤ 0.05. ( **c**) Pathway-enrichment analysis using ClueGO for differentially expressed up-regulated pathways in early testis compared to control samples (log _2_FC≥1, *P*-value≤0.005).

Other genes that are differentially expressed in the early testis compared to controls are shown in the heatmap in Figure 6c, Figure 7a and Figure 7b. These include the gene encoding renin ( *REN*) as well as mesonephric factors such as *NPHS2* and *WT1*. Pathway-enrichment analysis using ClueGO confirmed “sex differentiation” and “renal system development” as the two major up-regulated pathways in the dataset ( Figure 7c).

### Modelling dynamic changes in the testis downstream of SRY

**Figure 8.**
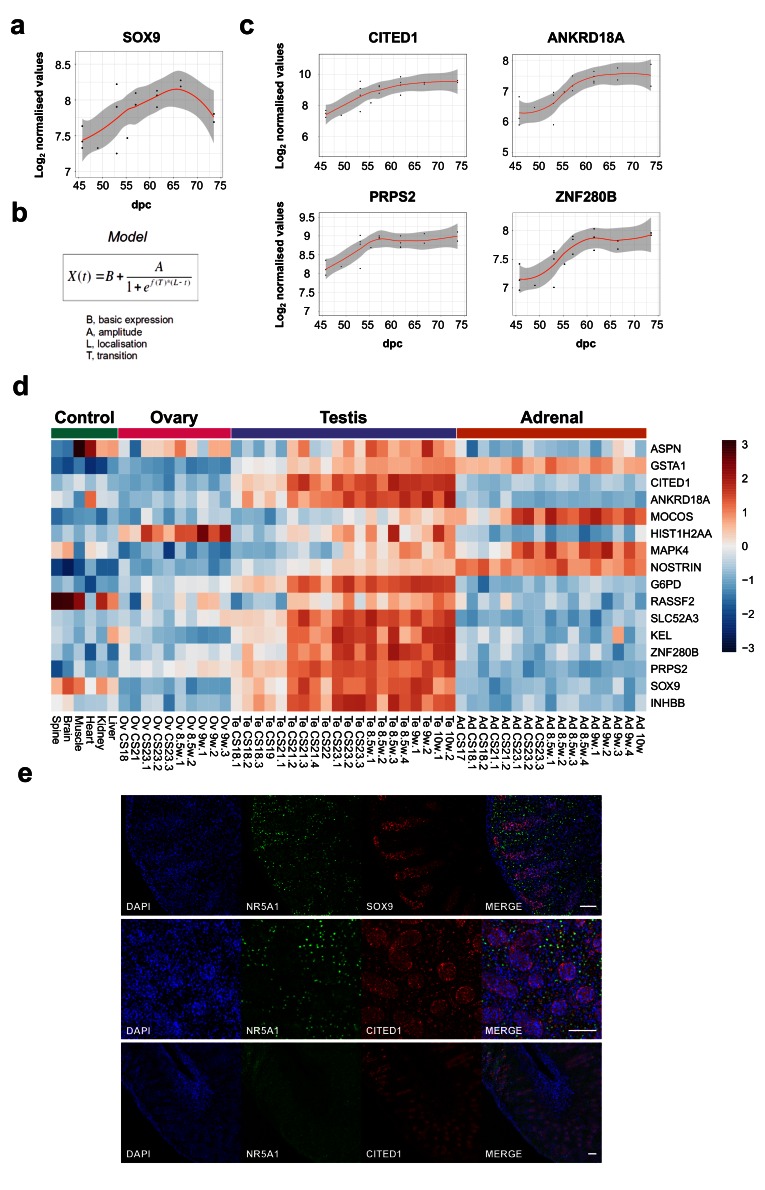
Identification of potential genes downstream of *SRY* using the BALT model. ( **a**) Scatter plot showing curvilinear upregulation of *SOX9* during testis development (45 to 74 dpc). Loess method was used for fitting a smooth curve between variables. ( **b**) Equation used in the BALT mathematical model (see methods for details). ( **c**) Scatter plots showing changes in gene expression patterns for *CITED1*, *ANKRD18A*, *PRPS2* and *ZNF280B* in the developing testis (45 to 74 dpc). Loess method was used for fitting a smooth curve between variables. ( **d**) Heatmap representing normalised gene expression values for genes showing similar expression patterns to *SOX9,* based on the BALT mathematical model. The intensity of gene expression is indicated by a colour scale: blue for lowest and red for highest expression levels. For all samples shown, *P*-value≤0.005. ( **e**) Immunohistochemistry for NR5A1 (SF-1, staining Leydig cells), SOX9, and CITED1 in the human fetal testis (11 wpc). Nuclei are counterstained blue with DAPI. Scale bars, 100 µm.

Studies in mice have shown that *SOX9* is a target of SRY, and disruption of *SOX9* in humans results in testicular dysgenesis ( Bashamboo *et al.*, 2017; Bishop *et al.*, 2000; Sekido & Lovell-Badge, 2008). However, the dynamics of SOX9 expression in human testis development are still not well understood and it is not established whether *SOX9* is the only potential SRY target ( Hanley *et al.*, 2000; Ostrer *et al.*, 2007). Analysis of *SOX9* across the testis series revealed an approximate 2-fold increase in gene expression levels with a curvilinear pattern ( Figure 8a).

In order to assess detailed patterns and changes in gene expression across the time series dataset, and to be able to group genes with similar dynamic expression patterns together, a phenomenological mathematical model was developed that allowed expression points of any gene to be described based on the basal gene expression value (“B”), the amplitude of the change (“A”), the localisation time of the maximum transition (“L”), and the rate of transition (“T”) (“BALT model”) ( Figure 8b) (See methods for details).

Using this approach, several genes were found to have similar expression dynamics to *SOX9* ( Figure 8c, Figure 8d, Table 2 and Supplementary Table 2). Analysis of the heatmap for these factors in the entire dataset showed that a subset of these genes had predominantly testis-specific upregulation ( *CITED1*, *ANKRD18A*, *G6PD*, *SLC52A3*, *KEL*, *ZNF280B*, *PRPS2*, *INHBB*) ( Figure 8d and Supplementary Figure 1). Immunohistochemistry of human fetal testis showed strong expression of CITED1 in seminiferous cords in a similar pattern to SOX9 ( Figure 8e). Similar expression data are seen for CITED1 in the adult testis ( CITED1 in the Human Protein Atlas). Adult expression data confirmed predominant testis expression of *ZNF280B* and *PRPS2*, whereas several other genes are expressed in adult testis but also strongly in at least one other system (e.g., *ANKRD18A*, *SLC52A3*, *KEL*) (Human Protein Atlas). In addition, *INHBB* encodes the β-subunit of inhibin B, a well-established testis protein, which together with *SOX9* provides validation for the dataset ( Valeri *et al.*, 2013).

**Table 2.** Potential biological functions of genes up-regulated during early testis development. Monogenic disorders associated with these factors are shown in italics. Potential function has been summarised from data in the GeneCards database.

Gene	Protein	Potential function
ASPN	Asporin	Leucine-rich repeat (LRR) protein associated with the cartilage matrix
GSTA1	Glutathione- S-transferase, alpha-1	Enzyme involved in detoxification of cellular compounds by conjugation with reduced glutathione
CITED1	CBP/p300-interacting transactivator with Glu/Asp-rich-C-terminal domain	Transcriptional co-regulator
ANKRD18A	Ankyrin repeat domain-containing protein 18A	Unknown
MOCOS	Molybdenum cofactor sulfurase	Enzyme involved in sulphuration of the molybdenum cofactor of xanthine dehydrogenase and aldehyde oxidase
HIST1H2AA	Histone gene cluster 1, H2A histone family, member A	Replication-dependent histone
MAPK4	Mitogen-activated protein kinase 4	Regulator of cell signalling
NOSTRIN	Nitric oxide synthase trafficker	Protein that binds endothelial nitric oxide synthase (eNOS) and triggers translocation of ENOS from the plasma membrane to sub-cellular structures
G6PD	Glucose-6-phosphate dehydrogenase	Enzyme involved in generation of NADPH in the hexose monophosphate pathway *(associated with G6PD* *deficiency)*
RASSF2	RAS association domain family protein 2	Protein that interacts with RAS proteins in cell growth and mitogenesis
SLC52A3	Solute carrier family 52, member 3	Transmembrane protein that mediates cellular uptake of riboflavin *(associated with Brown-Vialetto-van Laere* *syndrome type 1)*
KEL	Kell blood group metalloendopeptidase	Metalloendopeptidase that generates active endothelin-3 from big endothelin-3
ZNF280B	Zinc finger protein 280B	Transcription factor that upregulates expression of MDM2 and negatively regulates p53
PRPS2	Phosphoribosylpyrophosphate synthetase 2	Enzyme involved in regulation of nucleotide production pathways
SOX9	SRY-box 9	Transcription factor involved in the regulation of testis development
INHBB	Inhibin beta B	Growth and differentiation factor involved in reproduction and development

### Identifying known and novel components of steroidogenesis

**Figure 9.**
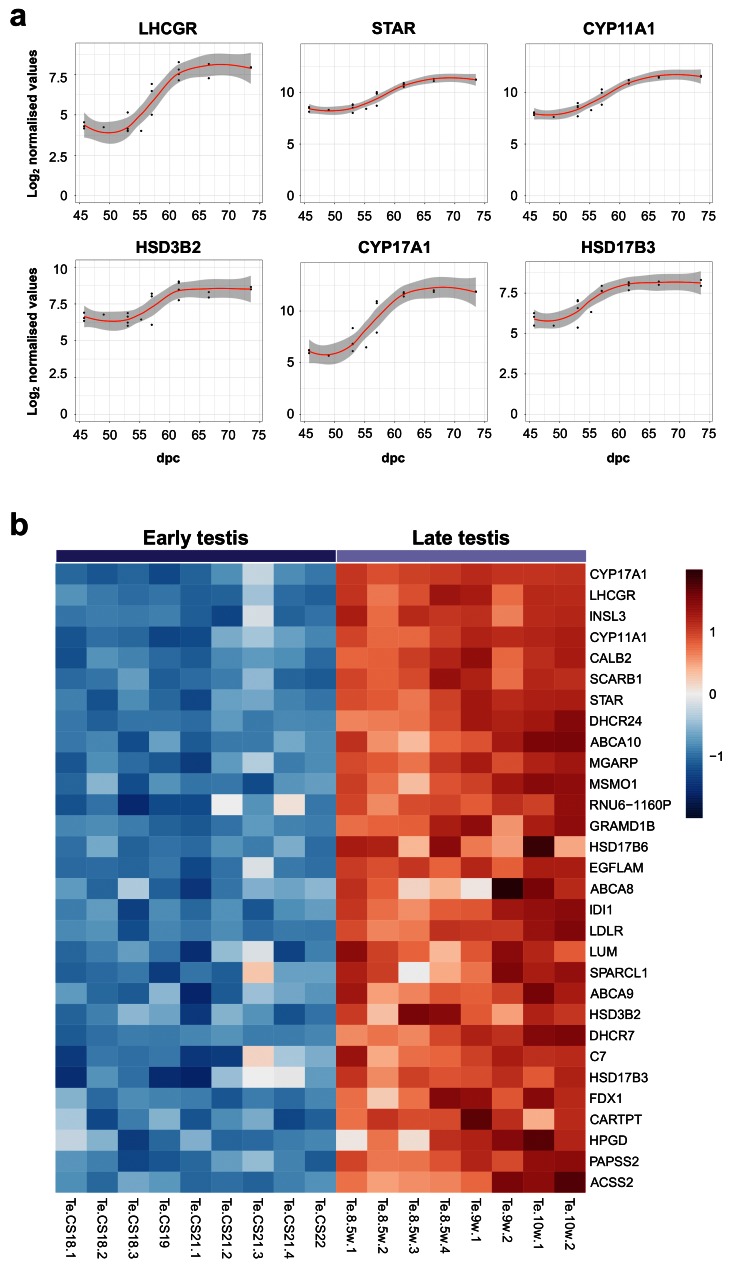
Determination of genes up-regulated with the onset of fetal testicular steroidogenesis. ( **a**) Scatter plots showing changes in gene expression patterns for known testicular steroidogenic genes, *LHCGR*, *STAR*, *CYP11A1*, *HSD3B2*, *CYP17A1* and *HSD17B3*. The plot shows normalised gene expression values for testis samples between approximately 46 and 74 dpc. Loess method was used for fitting a smooth curve between variables. ( **b**) Heatmap representing normalised gene expression values for the top 30 differentially up-regulated genes when comparing late (F1 to F3) ( *N*=8) and early (CS18 to CS22) ( *N*=9) testis samples. The intensity of gene expression is indicated by a colour scale: blue for lowest and red for highest expression levels. For all samples shown, *P*-value≤1e-4. Genes are ordered according to descending log _2_FC values.

**Figure 10.**
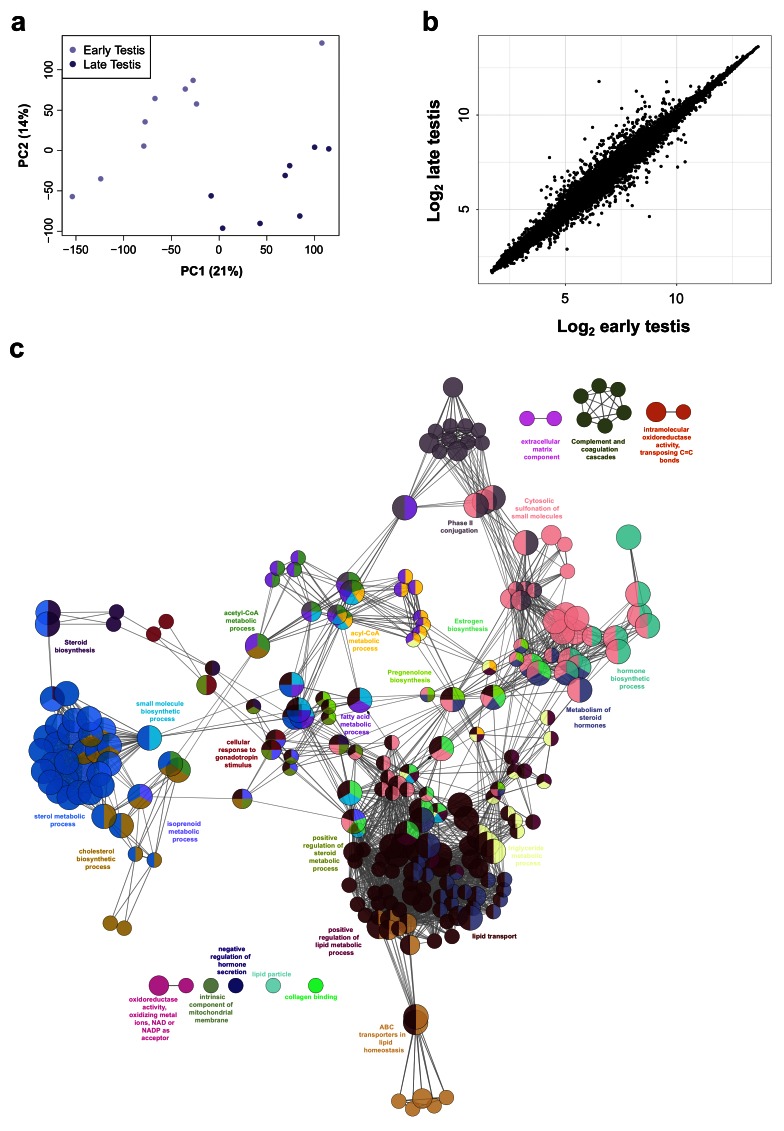
Differentially expressed genes in early compared to late testis samples. ( **a**) Principal component analysis of early ( *N*=9) compared to late ( *N*=8) testis samples. The scatter plot shows samples plotted on the two first principal components (PC1, PC2). ( **b**) Correlation plot for log _2 _mean gene expression levels in the early testis (CS18 to CS22) versus late testis (F1 to F3) samples (Pearson’s product-moment correlation test correlation coefficient=0.992, *P*-value< 2.2e-16). ( **c**) Pathway-enrichment analysis using ClueGO for differentially up-regulated genes in early testis compared to late testis samples (log _2_FC≥1, *P*-value≤0.01).

Using the “BALT” model across the time-series dataset, the expression of key known components of testis steroidogenesis showed marked upregulation around a fixed time point (“L”) between 54–57 dpc ( *LHCGR*, *STAR*, *CYP11A1*, *HSD3B2*, *CYP17A1*, *HSD17B3*) ( Figure 9a). Expression levels of all these enzymes showed a sigmoid-type pattern, suggesting that discrete biological events occur in this fixed window ( Supplementary Table 3). The heatmap of normalised gene expression plots for these enzymes showed similar discrete changes between early testis (CS18 to CS22) and late testis (F1 to F3) for many known components involved in testosterone synthesis ( Figure 9b). Principal components analysis also demonstrated segregation of samples into two distinct groups ( Figure 10a). A subset of genes was seen to be up-regulated with age in a correlation plot ( Figure 10b), and pathway-enrichment analysis strongly reflected processes involving “cholesterol”, “sterol” and “steroid” metabolism or biosynthesis ( Figure 10c). Therefore, CS23 (8 weeks) was a discrete point around which the onset of testicular steroidogenesis could be modelled, and samples either side of this time point were used for “pre-steroidogenesis” (“early”) versus “post-steroidogenesis” (“late”) comparisons. An overview of key genes in this dataset, many of which are associated with monogenic disorders in humans, is shown in Table 3.

**Table 3.** The top 20 differentially expressed genes after the onset of testicular steroidogenesis (“ *late testis” vs “early testis*”), with any associated clinical conditions in humans. Several other high-ranking genes associated with monogenic disorders are also shown. Genes are ranked based on log _2_FC in the late testis (n=8) compared to early testis (n=9). Further clinical information is available at Online Mendelian Inheritance in Man ( OMIM).

Rank	Gene	log _2_FC	Fold change	OMIM gene	Clinical condition (monogenic)
1	CYP17A1	5.27	38.5	609300	Congenital adrenal hyperplasia (due to 17 α-hydroxylase deficiency)
2	LHCGR	3.47	11.1	152790	Leydig cell hypoplasia
3	INSL3	3.13	8.8	146738	Cryptorchidism
4	CYP11A1	3.08	8.5	118485	Congenital adrenal hyperplasia (due to CYP11A1/ P450scc deficiency)
5	CALB2	2.86	7.3		
6	SCARB1	2.83	7.1	601040	High-density lipoprotein elevation
7	STAR	2.54	5.8	600617	Congenital lipoid adrenal hyperplasia; Familial glucocorticoid deficiency type 3
8	DHCR24	2.22	4.6	606418	Desmosterolosis
9	ABCA10	2.21	4.6		
10	MGARP	2.16	4.5		
11	MSMO1	2.12	4.3	607545	Microcephaly, congenital cataract and psoriasiform dermatitis
12	RNU6-1160P	2.11	4.3		
13	GRAMD1B	2.04	4.1		
14	HSD17B6	2.03	4.1		
15	EGFLAM	2.02	4.1		
16	ABCA8	2.02	4.0		
17	IDI1	2.01	4.0		
18	LDLR	1.95	3.9	600694	Familial hypercholesterolaemia
19	LUM	1.95	3.9		
20	SPARCL1	1.94	3.8		
22	HSD3B2	1.92	3.8	613890	Congenital adrenal hyperplasia (due to 3 β-hydroxsteroid dehydrogenase deficiency type 2)
23	DHCR7	1.92	3.8	602858	Smith-Lemli-Opitz syndrome
24	C7	1.87	3.7	217070	C7 (complement) deficiency
25	HSD17B3	1.87	3.7	605573	17 β-hydroxysteroid dehydrogenase deficiency type 3
28	HPGD	1.84	3.6	601688	Cranioosteoarthropathy; Primary hypertrophic osteoarthropathy type 1
29	PAPSS2	1.83	3.5	603005	Brachyolmia type 4 with mild epiphyseal and metaphyseal changes, adrenal hyperandrogenism
41	FDPS	1.61	3.0	134629	Porokeratosis type 9
42	MVD	1.59	3.0	603236	Porokeratosis type 7
49	NPC1	1.55	2.9	607623	Niemann-Pick disease type C1
68	INHA	1.40	2.6	147380	Adrenocortical carcinoma (paediatric)
100	AMH	1.18	2.3	600956	Persistent Müllerian duct syndrome, type 1
119	POR	1.07	2.1	124015	P450 oxidoreductase deficiency, Antley-Bixler syndrome

**Figure 11.**
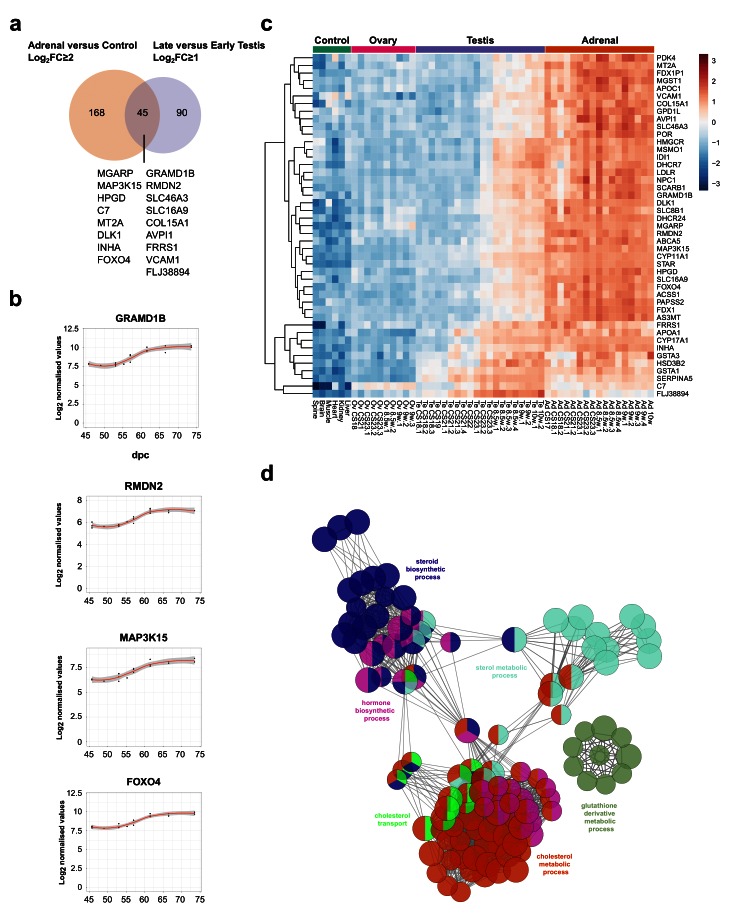
Identification of novel steroidogenesis genes. ( **a**) Venn diagram displaying the overlap for up-regulated genes between adrenal and control samples (log _2_FC≥2) with late versus early testis samples (log _2_FC≥1). A subset of genes found in the intersection are displayed, which are not yet established components of steroidogenesis. In all cases, *P*-value≤0.05. ( **b**) Scatter plots showing changes in gene expression patterns for four novel factors, *GRAMD1B, RMDN2*, *MAP3K15*, and *FOXO4*. The plot shows normalised gene expression values for testis samples between approximately 46 and 74 dpc. Loess method was used for fitting a smooth curve between variables. ( **c**) Heatmap representing normalised gene expression values for the 45 genes identified in the intersection between adrenal versus ** control and late testis versus early testis. The intensity of gene expression is indicated by a colour scale: blue for lowest and red for highest expression levels. Row-based unsupervised hierarchical clustering was performed. ( **d**) Pathway-enrichment analysis using ClueGO for the 45 genes identified in the intersection shown in ( **a**).

In order to identify potential novel core components of steroidogenesis in the adrenal gland and testis, genes were identified that were both up-regulated in late testis samples compared to early ones (post-steroidogenesis versus pre-steroidogenesis, log _2_FC≥1), and up-regulated in adrenal samples versus controls (adrenal versus controls, log _2_FC≥2) ( Figure 11a). A total of 45 overlapping genes were found, including all relevant known steroidogenic enzymes and 17 novel genes ( Figure 11a–c, Table 4). Enrichment-pathway analysis showed upregulation of genes involved in “cholesterol transport and metabolism” as well as “steroid biosynthesis”, further validating the analysis ( Figure 11d). The heatmap for these 45 genes confirmed highly selective expression in the fetal adrenal gland and in the fetal testis, but largely after CS23 ( Figure 11c).

**Table 4.** Known and potentially novel genes identified as components of steroidogenesis in humans. These 45 genes were determined from the overlap between genes differentially expressed in the adrenal gland vs. control (log _2_FC≥2) and in the testis vs. control (log _2_FC≥1).

**Steroidogenesis**
*CYP17A1, CYP11A1, STAR, FDX1P1, FDX1, PAPSS2, HSD3B2, POR*
**Cholesterol metabolism & oxidative stress**
*SERPINA5, SCARB1, DHCR24, APOA1, APOC1, MSMO1, AS3MT, LDLR, DHCR7,* *ACSS1, NPC1, PDK4, ABCA5, IDI1, GPD1L, HMGCR*
**Oxidative stress**
*GSTA1, MGST1, SLC8B1, GSTA3*
**Novel**
*MGARP (OSAP), MAP3K15, HPGD, C7, MT2A, DLK1 (PREF1), INHA, FOXO4,* *GRAMD1B, RMDN2, SLC46A3, SLC16A9, COL15A1, AVPI1, FRRS1, VCAM1,* *FLJ38894*

**Figure 12.**
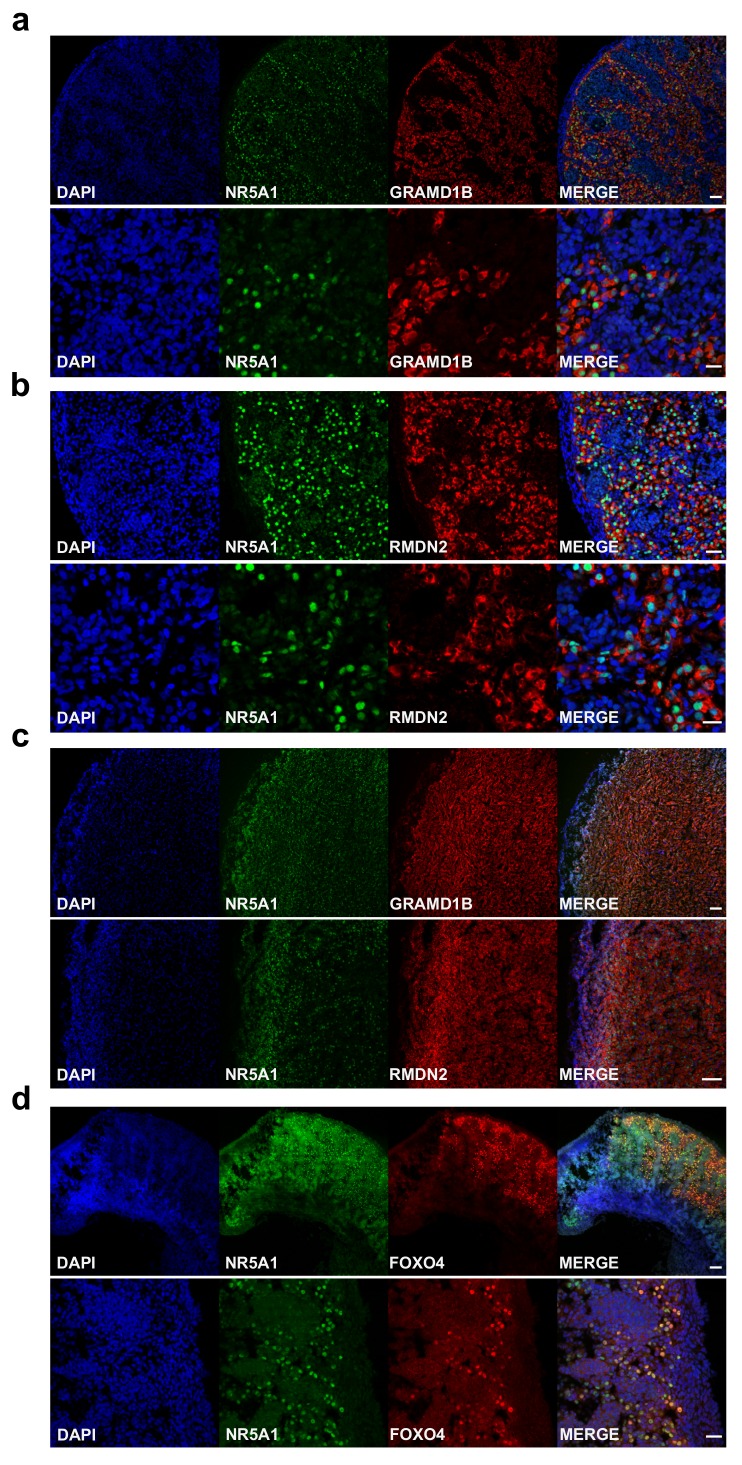
Validation of novel steroidogenic genes. ( **a**) Immunohistochemistry for GRAMD1B in human fetal testis at 9 wpc. NR5A1 (SF-1) was used to highlight Leydig cells (green). DAPI was used to counterstain nuclei (blue) and to highlight the outer capsule. Scale bars, 50 µm (top panels) and 20 µm (bottom panels). ( **b**) Immunohistochemistry of RMDN2 performed as above. Scale bars, 50 µm (top panels) and 20 µm (bottom panels). ( **c**) Immunohistochemistry of GRAMD1B and RMDN2 in the fetal adrenal gland at 9 wpc. NR5A1 (SF-1) was used to highlight the definitive zone and fetal zone cells (green). DAPI was used to counterstain nuclei (blue) and to highlight the outer capsule. Scale bars, 100 µm. ( **d**) Immunohistochemistry for FOXO4 in human fetal testis at 11 wpc. NR5A1 (SF-1) was used to highlight Leydig cells (green). DAP-I was used to counterstain nuclei (blue). Scale bar, 100 µm (top panels) and 20 µm (bottom panels).

An overview of the proposed function of the 17 potentially novel factors is shown in Table 5. These genes include *MGARP* (also known as *OSAP*), which may be involved in mitochondrial trafficking, *MAP3K15* ( *ASK3*) involved in cell signalling of stress, and the imprinted gene *DLK1* ( *PREF1*) implicated in growth and differentiation ( Guasti *et al.*, 2013; Naguro *et al.*, 2012; Zhou *et al.*, 2011). Two genes that have not been studied previously are *GRAMD1B* and *RMDN2* ( *FAM82A1*). These genes are clearly expressed with the onset of steroidogenesis ( Figure 11b,c) and show strong expression in the cytoplasm of NR5A1 (SF-1) positive fetal Leydig cells and adrenal cells by immunohistochemistry ( Figure 12a–c, Supplementary Figure 2a,b and Supplementary Figure 3a). However, currently the true biological function of these genes remains unknown. The transcription factor FOXO4 showed expression in the nuclei of Leydig cells in the fetal testis ( Figure 12d).

**Table 5.** Overview of potentially novel genes identified in human steroidogenesis. Potential function has been summarized from data in the GeneCards database, www.genecards.org.

Rank	Gene	Protein	OMIM gene	Potential function
1	MGARP (OSAP)	Mitochondria localized glutamic acid rich protein	-	Possible mitochondrial integrity and trafficking of mitochondria along microtubules
2	MAP3K15 (ASK3)	Mitogen-activated protein kinase kinase kinase 15	300820	Cell signalling of stress and apoptosis
3	HPGD	15-hydroxyprostaglandin dehydrogenase	601688	Metabolism and degradation of prostaglandins and NAD-dependent dehydrogenation
4	C7	Complement component 7	217070	Cytolytic phase of complement activation forming part of the membrane attack complex
5	MT2A	Metallothionein 2A	156360	Possible regulation of oxidative stress and binding to metal ions
6	DLK1 (PREF1, FA1)	Delta-like non-canonical notch ligand 1	176290	Imprinted (paternally expressed) regulator of cell growth and differentiation
7	INHA	Inhibin alpha	147380	Alpha subunit of TGF-beta glycoprotein hormone involved in regulation of hormone secretion, cell growth and apoptosis. Variants implicated in paediatric adrenal cancer.
8	FOXO4	Forkhead box O4	3000033	Transcription factor involved in oxidative stress, growth and differentiation, and leukaemia
9	GRAMD1B	GRAM domain containing 1B	(612559)	Locus has susceptibility to chronic lymphocytic leukaemia
10	RMDN2 (FAM82A1)	Regulator of microtubule dynamics 2	611872	Microtubule association during cell division
11	SLC46A3	Solute carrier family 46, member 3	616764	Membrane transport of small molecules
12	SLC16A9	Solute carrier family 16, member 9	614242	Monocarboxylic acid transporter across plasma membranes
13	COL15A1	Collagen type XV, alpha-1	120325	Structural component of muscle and vessels, possible precursor of restin (antiangiogenic)
14	AVPI1	Arginine vasopressin induced 1	-	May be involved in MAP kinase activation and cell cycling
15	FRRS1	Ferric chelate reductase 1	611578	Reduces ferric to ferrous iron before its transport from the endosome to cytoplasm
16	VCAM1	Vascular cell adhesion molecule 1	192225	Glycoprotein expressed in endothelial cells, interacts with integrins on leukocytes to mediate adhesion and signal transduction
17	FLJ38894	-	-	

### Identification of potential secreted proteins in the testis

**Figure 13.**
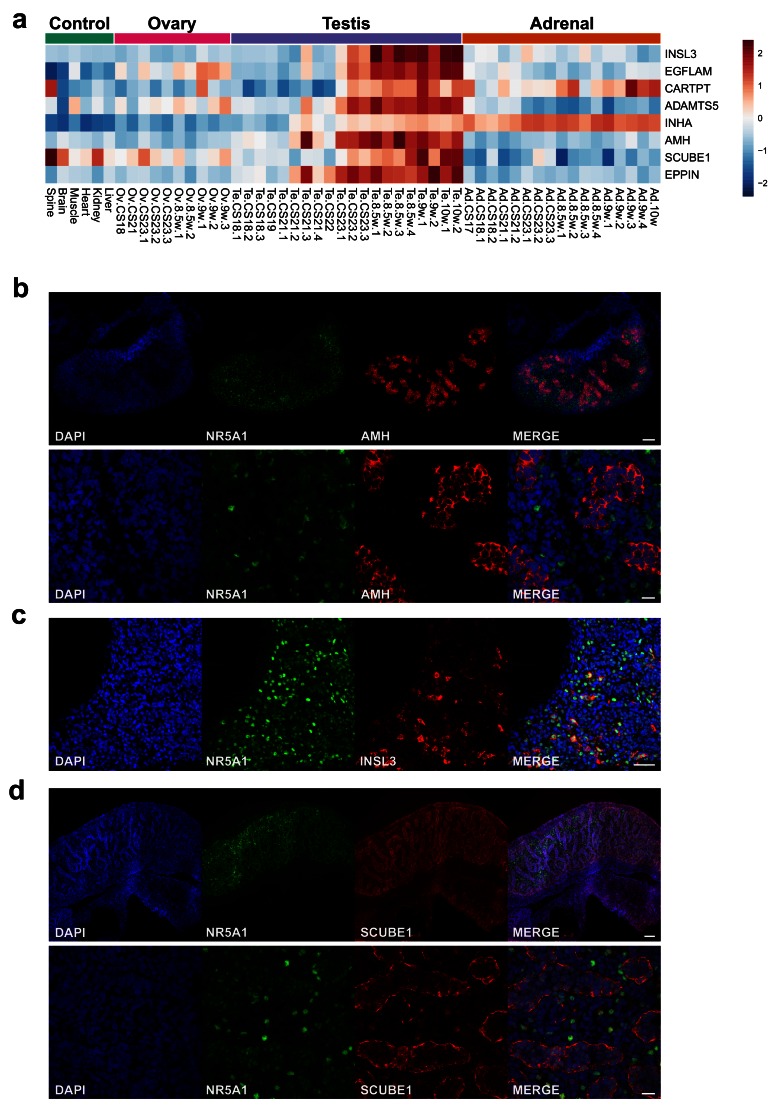
Identification of potential testis-secreted proteins. ( **a**) Heatmap representing normalised gene expression values for potential secreted proteins. The intensity of gene expression is indicated by a colour scale: blue for lowest and red for highest expression levels. ( **b**) Immunohistochemistry for AMH (MIS) in human fetal testis at 9 wpc. NR5A1 (SF-1) was used to highlight Leydig cells (green). DAPI was used to counterstain nuclei (blue) and to highlight the outer capsule. Scale bars, 100 µm (top panels) and 20 µm (bottom panels) ( **c**) Immunohistochemistry for INSL3 in human fetal testis at 9 wpc. NR5A1 (SF-1) was used to highlight Leydig cells (green). DAPI was used to counterstain nuclei (blue). Scale bar, 50 µm ( **d**) Immunohistochemistry for SCUBE1 in human fetal testis at 9 wpc. NR5A1 (SF-1) was used to highlight Leydig cells (green). DAPI was used to counterstain nuclei (blue). Scale bars, 100 µm (top panels) and 20 µm (bottom panels).

The analysis of genes that showed progressive upregulation in the developing testis was extended to try to identify novel biomarkers. The protein structures encoded by genes that were up-regulated in late testis samples (F1 to F3; FC≥1, Dataset 6) were reviewed for characteristics of secreted proteins, such as the presence of a cleaved signal peptide. Using this approach, the three major testis biomarkers Anti-Müllerian Hormone ( *AMH/MIS*), inhibin α-subunit ( *INHA*) and insulin-like 3 ( *INSL3*) were identified ( Figure 13a–c). Novel factors identified included EGFLAM, CARTPT, ADAMTS5, SCUBE1 and EPPIN ( Figure 13a). Expression of SCUBE1 was shown in seminiferous cords in the developing testis ( Figure 13d).

### Ovarian development is not a passive process

Although ovary development was once thought to be a less biologically active process, several studies in the mouse have shown changes in discrete sets of differentially expressed genes in the early ovary ( Beverdam & Koopman, 2006; Jameson *et al.*, 2012; Nef *et al.*, 2005). However, it is unclear whether similar effects are also present during early human gonad development.

**Figure 14.**
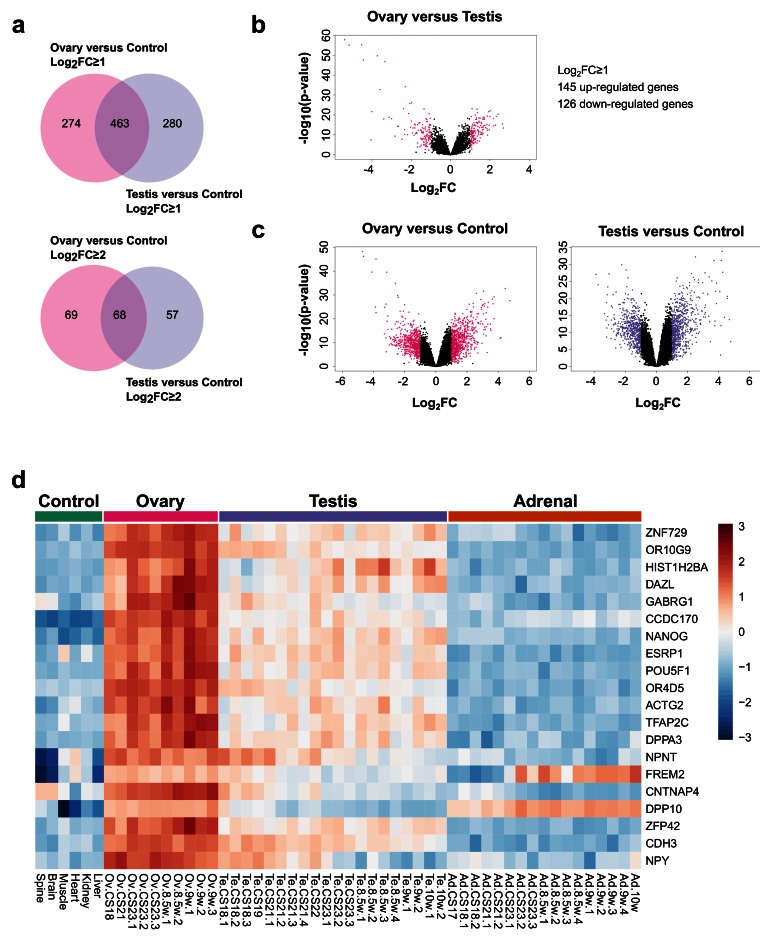
Ovary development dynamics. ( **a**) Venn diagram displaying the overlap for up-regulated genes between ovary and control versus testis and control samples using log _2_FC≥1 (upper panel) and log _2_FC≥2 (lower panel) cut-offs. *P*-value≤0.05. ( **b**) Volcano plot analysis of differentially expressed genes in ovary versus testis samples. Genes with an absolute log _2_FC≥1 and *P*-value≤0.05 are shown in pink. ( **c**) Volcano plot analysis of differentially expressed genes in the ovary versus control samples (left panel) and testis versus control samples (right panel). Genes with an absolute log _2_FC≥1 and *P*-value≤0.05 are shown in pink and purple respectively. ( **d**) Heatmap representing normalised gene expression values for the top 20 differentially expressed genes found only in the ovary versus control samples, using a log _2_FC≥2 cut-off. The intensity of gene expression is indicated by a colour scale: blue for lowest and red for highest expression levels. *P*-value≤1e-5. Genes are ordered according to decreasing log _2_FC values.

By studying up-regulated genes in the ovary and comparing them to control samples, ( Dataset 4) and by studying up-regulated genes in the testis and comparing them to controls ( Dataset 3), very similar numbers of gonad-specific differentially expressed genes were found (log _2_FC≥ 1, ovary 274 versus testis 280; log _2_FC≥2, ovary 69 versus testis 57) ( Figure 14a). As expected, there was substantial overlap in genes that were co-expressed in both the ovary and the testis (log _2_FC≥1, 463; log _2_FC≥2, 68). Although the number of strongly up-regulated genes was somewhat higher in the testis compared to the ovary when observed in a volcano plot ( Figure 14b and Dataset 7), in part due to Y chromosomal genes, a similar scatter of up- and down-regulated genes was seen for the ovary versus control as for the testis versus control, again reinforcing the concept of the developing ovary having discrete genetic activity ( Figure 14c).

**Figure 15.**
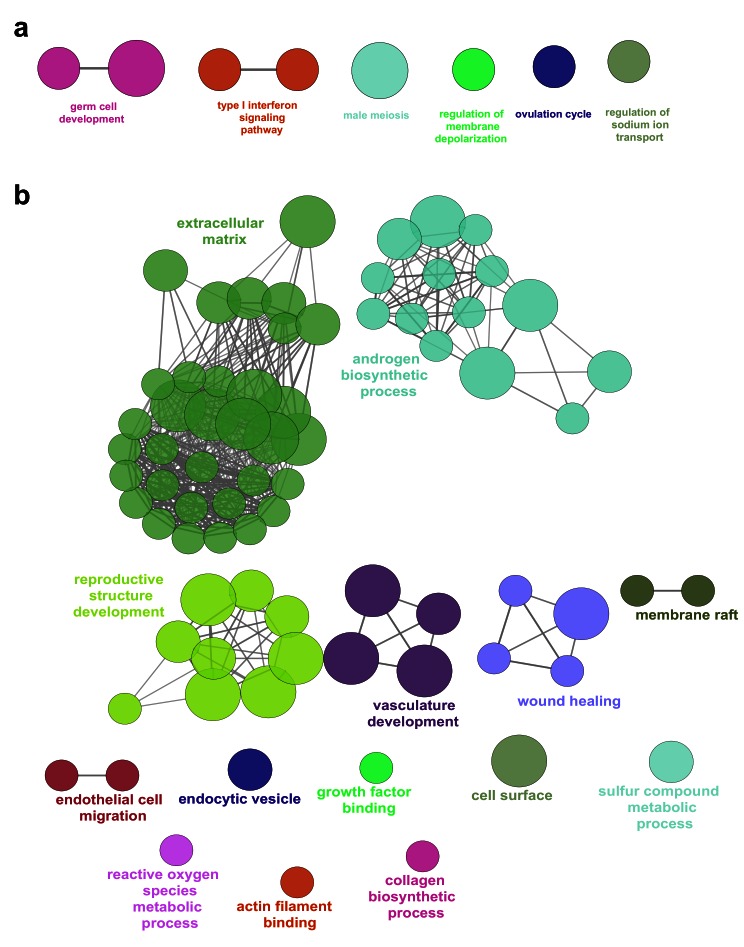
Pathway enrichment analysis of ovary-specific genes. ( **a**) Pathway-enrichment analysis using ClueGO for differentially up-regulated genes in the ovary compared to control, with testis up-regulated genes excluded (log _2_FC≥1, *P*-value≤0.05). ( **b**) Pathway-enrichment analysis using ClueGO for differentially up-regulated genes in the testis compared to control, with ovary up-regulated genes excluded (log _2_FC≥1, *P*-value≤0.05).

Analysis of the top 20 up-regulated genes in the ovary compared to control data is shown in the heatmap in Figure 14d. Many of these genes were also expressed to some extent in the testis, and may represent factors involved in pluripotency and germ cell development such as HIST1H2BA, NANOG and POU5F1 (also known as OCT4). Expression of several genes appeared to increase in the ovary across the time course (CS18 to F2) and to decrease in the testis, such as *OR10G9*, *GABRG1*, *OR4D5*, *NPNT*, *CNTNAP4* and *NPY* ( Figure 14d). These genes could potentially encode novel components of an ovary-specification program. However, pathway-enrichment analysis of the 274 genes differentially up-regulated in the ovary compared to controls (log _2_FC≥1, *P*<0.05) resulted in very few biological processes other than “germ cell development” and “type 1 interferon signalling pathway” ( Figure 15a). In contrast, pathway-enrichment analysis for the 280 genes up-regulated in the testis samples versus controls (log _2_FC≥1, *P*<0.05) revealed many more detailed networks, with enrichment in terms such as “androgen” and “reproductive” processes ( Figure 15b). Taken together, these data suggest that current knowledge of the genetic events in the early stages of human ovary development is still very limited.

## Discussion

Adrenal development and gonad development are two of the most fundamental biological processes. However, current knowledge about the genetic mechanisms underlying these events is derived largely from studies in mice ( Beverdam & Koopman, 2006; Inoue *et al.*, 2016; Jameson *et al.*, 2012; McClelland *et al.*, 2015; McClelland & Yao, 2017; McDowell *et al.*, 2012; Nef *et al.*, 2005; Xing *et al.*, 2015) and few data are currently available from humans ( Fowler *et al.*, 2009; Gerrard *et al.*, 2016; Houmard *et al.*, 2009; O’Shaughnessy *et al.*, 2007), especially during the first trimester or across all three tissues. Of the published data, the recent study from Gerrard *et al*. provides the most detailed insight into early human adrenal and testis expressed genes currently available, but this work included just two samples from pooled RNA between CS18 and CS22 ( Gerrard *et al.*, 2016). Given the potential biological differences between species, as well as a lack of detailed time course data for all three tissues, our aim was to generate a detailed “Atlas” of genomic events across a critical period in human embryonic and fetal development (6 to 10 wpc).

Initial analysis focused on global gene expression patterns in the adrenal, testis and ovary compared to control tissues. Control samples were chosen to represent a range of developmental tissues (neuroectoderm, mesoderm, endoderm) across the study period. Approximately 0.5–1% of all genes were differentially expressed in each tissue at log _2_FC≥2 (absolute fold change 4) and 3–5% of all genes at log _2_FC≥1 (absolute fold change 2). The strong overlap between the adrenal and testis partly reflected shared components of steroidogenesis, whereas the overlap between testis and ovary reflected the shared origins of these tissues as well as the presence of germ cells.

Initial validation of our dataset came from known genes involved in distinct biological processes or monogenic disorders in humans. For example, two of the most strongly expressed transcription factors were the nuclear receptors *NR0B1* (DAX-1), which was found in the adrenal and testis, and *NR5A1* (SF-1), which was found in all three tissues. Pathogenic variants in *NR0B1* cause X-linked adrenal hypoplasia and impaired spermatogenesis in males ( Suntharalingham *et al.*, 2015), whereas pathogenic variants in *NR5A1* are associated with adrenal insufficiency, testicular dysfunction and primary ovarian insufficiency ( Achermann *et al.*, 1999; Lourenço *et al.*, 2009; McElreavey & Achermann, 2017; Suntharalingham *et al.*, 2015). These findings, together with the identification and distribution of steroidogenic components and the detection of known gonadal regulators (e.g. *EMX2*, *LHX9*, *TCF21*, *WT1*, *GATA4*) and germ cell pluripotency factors (e.g *POU5F1*, *NANOG*) provided strong validation for our dataset ( Svingen & Koopman, 2013).

Analysis of genes that were differentially expressed in the adrenal gland provided further support for our experimental approach. The six genes most strongly expressed all represent major core components of adrenal steroidogenesis ( *CYP17A1*, *CYP11A1*, *SULT2A1*, *STAR*, *MC2R*, *CYP11B1*) ( Miller & Auchus, 2011). All have associations with human adrenal disorders except for sulfotransferase 2A1, which is involved in sulphation of dehydroepiandrosterone (DHEA) to dehydroepiandrosterone sulphate (DHEAS), the main steroid synthesised by the fetal adrenal zone. Novel factors identified in the adrenal gland include adrenal-specific factors such as ASB4 and NPR3, and two kinases (MAP3K15, NRK) that are also expressed in the testis. FOXO4 and TBX3 emerged as novel transcription factors that have not previously been implicated in adrenal development. Of note, several genes associated with adrenal insufficiency in humans were not differentially expressed during early development; namely *NNT*, *TXNRD2*, *AAAS*, *MCM4* and *SGPL1*. Most of these factors have ubiquitous expression and may have a pathogenic effect through oxidative stress pathways or through the accumulation of toxic metabolites. These conditions may be precipitated by postnatal stress and affected children very rarely present with adrenal insufficiency in early infancy, unlike other conditions associated with differentially expressed genes where children often present in the neonatal period (e.g. *NR0B1*, *NR5A1, SAMD9*, *CDKN1C*, *MRAP*, *MC2R*, *STAR*, *CYP11A1*, *HSD3B2*, *CYP17A1*, *POR*, *CYP21A2*, *CYP11B1*) ( Guran *et al.*, 2016).

The role of *SRY* as the primary testis-determining gene has been known for 25 years, following transgenic mouse studies as well as the discovery of duplication of *SRY* in individuals with 46,XX testicular disorders of sex development (DSD) or disruption of *SRY* in 46,XY testicular dysgenesis ( Bashamboo *et al.*, 2017; Koopman *et al.*, 2016; Svingen & Koopman, 2013). *SRY* is believed to undergo a transient wave of expression in the developing testis that regulates transcription of downstream factors such as *SOX9* and initiates testis development pathways ( Hiramatsu *et al.*, 2009). Although *in situ* hybridization studies of *SRY* in early human testis have been reported, suggesting onset of *SRY* expression at approximately 42 dpc, data regarding *SRY* expression in humans are still very limited ( Hanley *et al.*, 2000). *SRY* is already up-regulated at the start of our study (45 dpc) and falls with the onset of steroidogenesis around 56dpc. By comparing early testis samples (CS18–CS22) with 46,XY controls, *SRY* was the only protein coding Y-chromosome gene detected. Although it is widely assumed that SRY is the only Y-chromosomal testis-determining factor, this provides the first direct evidence in humans that this is the case.

Other genes up-regulated in the early testis show an overlap with mesonephric development and factors involved in nephrotic syndrome (e.g., *NPHS2*, *WT1*). Of note, the highest differentially expressed gene encodes renin ( *REN*), which supports data from previous studies of mouse testis development ( Beverdam & Koopman, 2006; Bouma *et al.*, 2007; Inoue *et al.*, 2016; Nef *et al.*, 2005). Renin is a hormone typically secreted from the juxtaglomerular apparatus in the kidney that regulates angiotensin and aldosterone, thereby modulating renal sodium reabsorption and arterial blood pressure. Its role in the testis is unclear, as is its expression in extra-adrenal tissue. Furthermore, it has been proposed that SRY-dependent expression of renin-angiotensin system genes, including *REN* itself, is a major factor responsible for differences in blood pressure between men and women ( Araujo *et al.*, 2015; Prokop *et al.*, 2012).

It is well-established that *SOX9* is a target of SRY in testis determination, and *SOX9* expression rises in the early human testis following expression of SRY ( Hanley *et al.*, 2000; Ostrer *et al.*, 2007; Sekido & Lovell-Badge, 2008). Although data from mice and humans suggest that SOX9 is in itself sufficient to trigger downstream testis pathways ( Bishop *et al.*, 2000; Kim *et al.*, 2015) it is unclear if other SRY targets exist, or what direct targets of SOX9 might be. Attempts have been made to investigate this using ChIP-Chip in mice, but this approach is challenging given the amount of fetal tissue needed at the correct stage of development ( Bhandari *et al.*, 2012; Li *et al.*, 2014).

An alternative approach is to study the pattern of *SOX9* expression across a time-series, and to identify other genes with similar expression profiles. Rather than using a correlative approach, we developed a mathematical model that could be used to capture subtle changes in our dataset more quantitatively. Using this approach, coupled with subsequent review of gene expression in different fetal and adult tissues, we have identified a small group of genes that are up-regulated with SOX9 in the testis (e.g., *CITED1*, *ZNF280B*, *PRPS2*).

Of these, CITED1 (encoding Cbp/p300 interacting transactivator with Glu/Asp rich carboxy-terminal domain 1) is of greatest interest, as it is a transcriptional co-regulator, and strongly expressed in the adult testis, epididymis and pituitary gland. *Cited1* knockout mice have growth restriction, late placental insufficiency and abnormalities in nephron patterning and pubertal mammary ductal morphogenesis ( Howlin *et al.*, 2006; Novitskaya *et al.*, 2011; Plisov, 2005; Rodriguez *et al.*, 2004). Although *Cited1* was identified as a target of Sry in one mouse ChIP-CHIP study ( Li *et al.*, 2014), little is known about *Cited1* in mouse testis. In contrast, the related protein Cited2 influences adrenal and gonad development in the mouse, potentially through the regulation of *Sf1* as well as of *Sry* itself ( Buaas *et al.*, 2009; Combes *et al.*, 2010). *CITED2* is ubiquitously expressed in humans and does not show differential expression in our datasets, and pathogenic variants in *CITED2* have not been found in patients with adrenal and adrenogonadal disorders, suggesting that CITED1 might be a more important co-regulator in humans ( Ferraz-de-Souza *et al.*, 2009). Amongst the other key genes identified, *ZNF280B* has been proposed to encode a negative regulator of p53 in prostate cancer cells ( Gao *et al.*, 2013), whereas *PRPS2* encodes a testis specific phosphoribosylpyrophosphate synthetase that may function as an anti-apoptotic factor ( Lei *et al.*, 2015; Taira *et al.*, 1990).

The advantage of having multiple serial samples for the testis is that the BALT model could be used to accurately pinpoint the onset of steroidogenesis. Using this approach a distinct sigmoid-shaped upregulation of all the known genes involved in testicular steroidogenesis was seen between 54 and 57 dpc, with marked increases in fold-change. This discrete change in activity allowed us to divide the datasets into “pre-steroidogenic” and “post-steroidogenic” stages.

In order to identify novel components involved in steroidogenesis, genes were identified that were up-regulated in the testis post-steroidogenesis compared to pre-steroidogenesis, and also up-regulated in the adrenal gland compared to control samples. A total of 45 genes were identified, including all known core components of steroidogenesis and many factors involved in cholesterol biosynthesis and oxidative stress.

The 17 “novel” genes identified included *MGARP* (also known as *OSAP*), *DLK1* ( *PREF1*), and *MAP3K15* ( *ASK3*). *MGARP* has been implicated in mitochondrial trafficking and steroidogenesis ( Jia *et al.*, 2014; Jin *et al.*, 2012; Zhou *et al.*, 2011), but may also be a key target of OCT4 during reprogramming to pluripotency ( Tiemann *et al.*, 2014). *DLK1* is an imprinted gene that is involved in inhibiting adipocyte differentiation, which has previously been shown to be expressed in the outer undifferentiated zone of the male rat adrenal gland and signals to the adrenal capsule to regulate zonation ( Guasti *et al.*, 2013). MAP3K15 seems to be a novel kinase linked to steroidogenesis, which has very recently been shown to be differentially expressed in the adult adrenal gland ( Bergman *et al.*, 2017). Of note, the *Map3k15/Ask3* knockout mouse is hypertensive and studies have linked this kinase to the response to osmotic stress and blood pressure regulation in the mouse kidney, through interactions with WNK1 or WNK4 ( Maruyama *et al.*, 2016; Naguro *et al.*, 2012). In our dataset, *MAP3K15* was predominantly expressed in steroidogenic tissue, and not in the kidney. Similar findings are seen in RNA expression data for *MAP3K15* in the Human Protein Atlas. Taken together, these findings suggest that adrenal effects of MAP3K15 may be an important area to focus future research on.

Other potential novel components of steroidogenesis with unknown function include *FOXO4*, *GRAMD1B* and *RMDN2* ( *FAM82A1*). FOXO transcription factors play important roles in development, cancer and metabolic homeostasis ( Coomans de Brachène & Demoulin, 2016). FOXO4 has been implicated as a negative regulator of cell proliferation in several tumours, but a link to steroidogenesis has not previously been established ( Li *et al.*, 2016). *GRAMD1B* has been shown to be enriched in mouse fetal Leydig cells following CRHR1 agonist stimulation ( McDowell *et al.*, 2012), and has been identified in other expression studies of mouse fetal Leydig cells ( Inoue *et al.*, 2016; McClelland & Yao, 2017). We have shown by immunohistochemistry that there is clear upregulation of GRAMD1B in the cytoplasm of NR5A1-positive steroidogenic cells with the onset of steroidogenesis in humans. Similar upregulation of expression of RMDN2, a putative microtubule regulator, was also seen in these cells. However, the exact functional role of both GRAMD1B and RMDN2 in steroidogenesis is currently unknown.

In addition to steroidogenesis, the fetal testis has important biological roles through the secretion of proteins or hormones that regulate Müllerian regression (e.g., AMH), endocrine feedback (e.g., inhibin B) and testis descent (e.g. INSL3) ( Valeri *et al.*, 2013). These circulating proteins are also proving to be important biomarkers of testis integrity and function postnatally. Using a strategy to identify genes encoding secreted proteins, all three major factors were identified, namely AMH, inhibin (α-subunit) and INSL3. Potential novel secreted proteins include EPPIN, which has been extensively studied as a novel approach to male contraception ( O’Rand *et al.*, 2016), and SCUBE1, a secreted cell surface glycoprotein belonging to the EGF superfamily. SCUBE1 was first identified from vascular endothelial cells and is released by activated platelets. Plasma levels of SCUBE1 have been measured as a potential biomarker in several conditions, such as gastric cancer, hypertension, ischaemic heart disease, and stroke ( Özkan *et al.*, 2013). Higher levels of SCUBE1 have been reported in adult males compared to females in some, but not all studies, although few control data exist ( Ulusoy *et al.*, 2012). An acute elevation in SCUBE1 has been demonstrated in experimental models of rat testis torsion, but it is unclear whether testicular SCUBE1 contributes significantly to circulating levels of SCUBE1 in humans ( Turedi *et al.*, 2015).

Although ovary development was originally viewed as a relatively passive process compared to testis development, studies in mice have shown that a similar number of differentially expressed genes are up-regulated in both organs during early development ( Beverdam & Koopman, 2006; Bouma *et al.*, 2007; Nef *et al.*, 2005). Our data show that this is true in human gonad development too, with 274 and 280 genes differentially expressed in the ovary and testis, respectively, at log _2_FC>1, and 69 and 57 genes at log _2_FC>2. However, pathway-enrichment analysis of the tissue-specific genes revealed virtually no annotated pathways in the ovary, whereas tissue-specific pathways were more abundant in the testis. As expected, amongst the differentially-expressed ovary factors are several key genes involved in germ cell development, e.g. *NANOG*, *POU5F1*, *TFAP2C*, *ZFP42* ( *REX1*). In addition, several genes involved in neurotransmission were higher in the developing ovary but were attenuated in the developing testis ( *OR10G9*, *GABRG1*, *OR4D5*, *NPNT, CNTNAP4* and *NPY*). These include two olfactory receptors of unknown function, a GABA receptor isoform subunit, neuropeptide Y and contactin associated protein like 4 (CNTNAP4), which has recently been implicated in GABAergic synaptic transmission ( Karayannis *et al.*, 2014). Clearly, our understanding of very early human ovary development is still limited.

Although we feel this is an important study in human development, there are several limitations. Firstly, whole tissue samples were used for RNA extraction and RNA analysis. Using this approach transcriptomic signals from small populations of cells might be relatively attenuated. For example, the interstitial/Leydig cell population in the testis is only a relatively small population of the total number of cells. We have addressed this by studying relative changes in gene expression over time, which is independent of the total signal strength, and confirming key findings by immunohistochemistry, although the proportion of cell populations many also change with time. Future studies of single cell transcriptomics will be able to address this in more detail. Secondly, these studies use microarray analysis rather than RNA-Seq. Microarray analyses are restricted by the specificity and number of probes and the arrays used in this study did not address differently spliced isoforms. Future studies using RNA-Seq might address both these points.

## Conclusion

We have developed an important dataset describing the genomic landscape across three different tissues and spanning critical stages in human embryonic and fetal development. We have identified several important elements previously determined from studies in mice (e.g. Renin in testis), but also several novel aspects that may be specific to humans (e.g. CITED1 rather than CITED2 in early testis). By studying time-series changes and differential expression patterns, we have discovered novel components of these important biological processes and interesting new pathways for further investigation. The dataset is highly validated based on known human disorders of adrenal and gonad function and will provide important insight to assign likely biological significance to new genes identified in whole exome and whole genome studies of patients with undiagnosed conditions. Finally, from the limited number of examples discussed above, novel insights from our genomic atlas could fuel many aspects of translational research, including biological sex-differences in humans, vulnerability to early life insults and programming, blood pressure regulation and stem cell/germ cell development.

## Data availability

The data referenced by this article are under copyright with the following copyright statement: Copyright: © 2017 del Valle I et al.

Array data are available from the ArrayExpress database under the accession number E-MTAB-5525.

Datasets are available from Open Science Framework ( OSF), with the DOI 10.17605/OSF.IO/WV5DA. ( Achermann, 2017).

**Dataset 1. Complete dataset of normalised log _2_ gene expression levels of all 53 samples included in the study.**

**Dataset 2. Differentially expressed genes in the adrenal gland compared to controls.**

**Dataset 3. Differentially expressed genes in the testis compared to controls.**

**Dataset 4. Differentially expressed genes in the ovary compared to controls.**

**Dataset 5. Differentially expressed genes in the early testis compared to controls.**

**Dataset 6. Differentially expressed genes in the late testis compared to early testis.**

**Dataset 7. Differentially expressed genes in the testis compared to ovary.**

## Consent

Human embryonic and fetal tissue samples used in this study were obtained in collaboration with the MRC/Wellcome Trust-funded Human Developmental Biology Resource ( HDBR). The HDBR is a tissue bank regulated by the Human Tissue Authority. Samples were collected with appropriate maternal written consent and with approval from the NRES Committee London-Fulham (REC reference 08/H0712/34+5).

## Supplementary material

**Supplementary Figure 1. Upregulation of novel testis genes over time.**

**Supplementary Figure 2. Upregulation of potentially novel steroidogenic genes in human testis over time.**

**Supplementary Figure 3. Expression of novel human steroidogenic genes in the adrenal gland.**

**Supplementary Table 1. Overview of all 53 tissue samples included in the study.**

**Supplementary Table 2. Genes that are up-regulated during early testis development with a similar pattern to SOX9, ranked by amplitude of change from basal expression.**

**Supplementary Table 3. Genes that are up-regulated around the onset of testicular steroidogenesis.**
