# Supplementary figures and images for: A genomic atlas of human adrenal and gonad development
Source: Wellcome Open Res. 2017 Oct 23;2:25. Originally published 2017 Apr 7. [Version 2] doi: 10.12688/wellcomeopenres.11253.2 (PMC5407452; doi:10.12688/wellcomeopenres.11253.2)

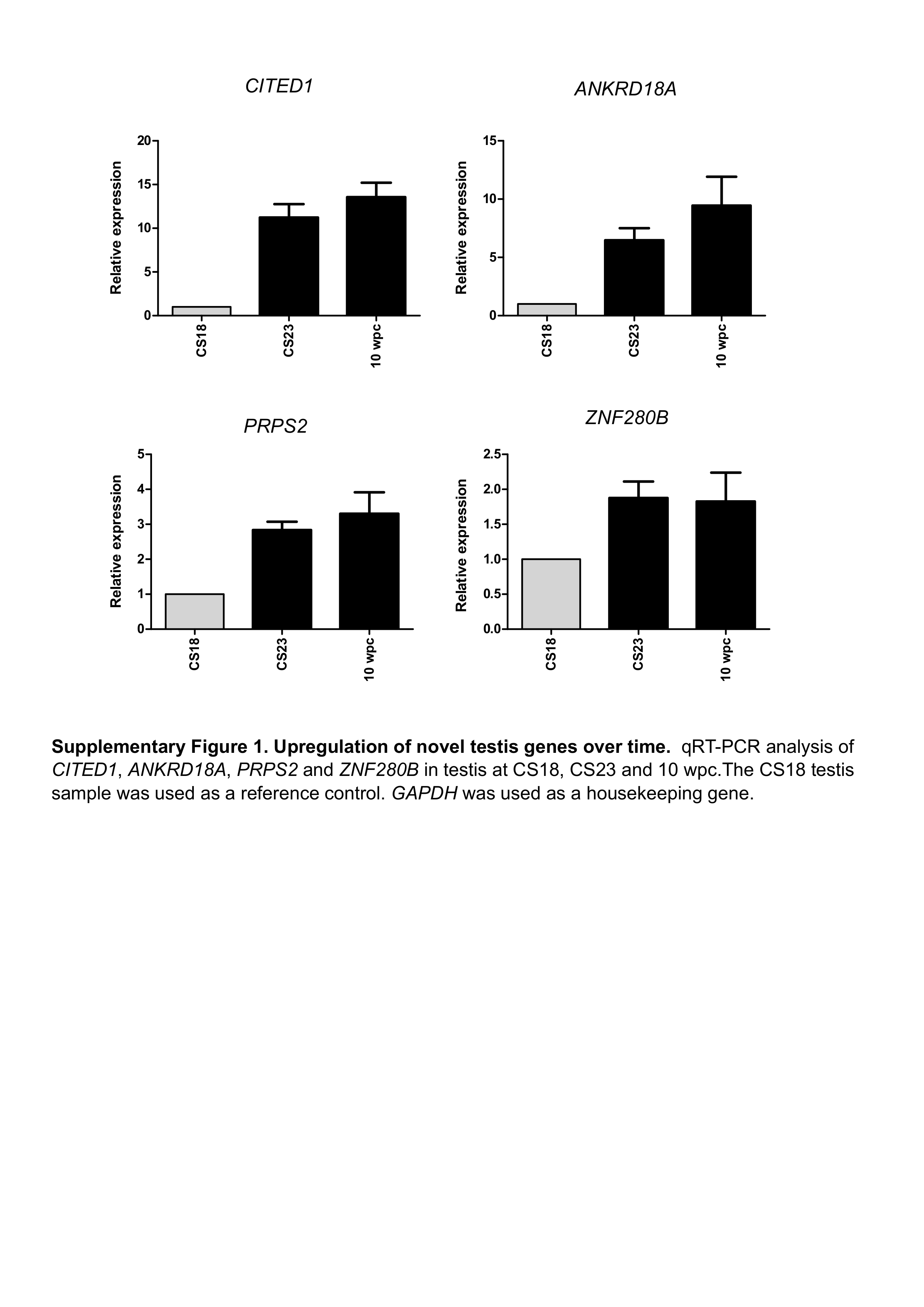

Supplement: Supplementary file 1 [file wellcomeopenres-2-14044-s0000.tgz › 8488d5d7-8e06-452e-b54a-3e5f4404ac8e.tiff]

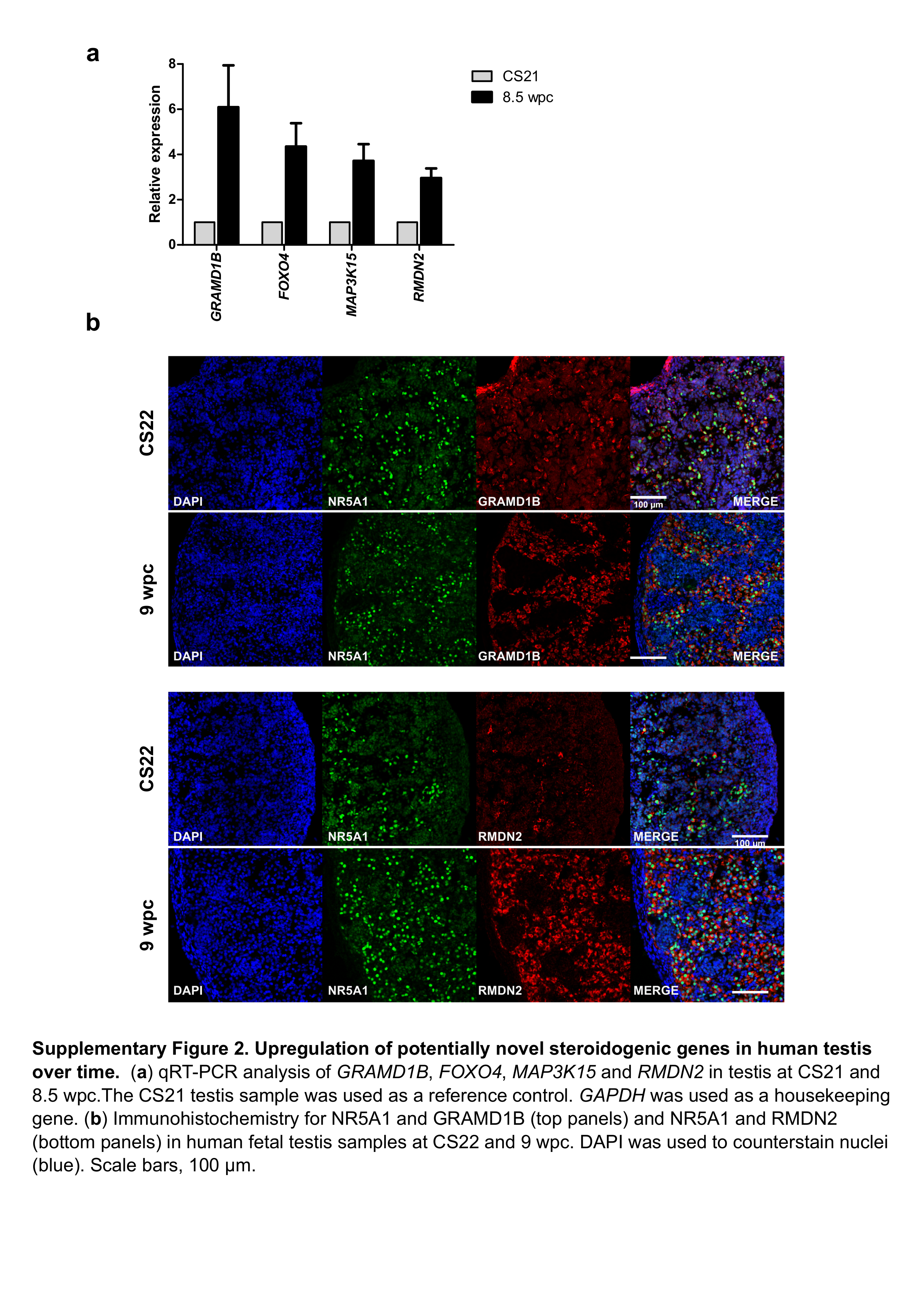

Supplement: Supplementary file 2 [file wellcomeopenres-2-14044-s0001.tgz › 1ba12e7c-5fab-445f-9916-6749ae6910ef.tiff]

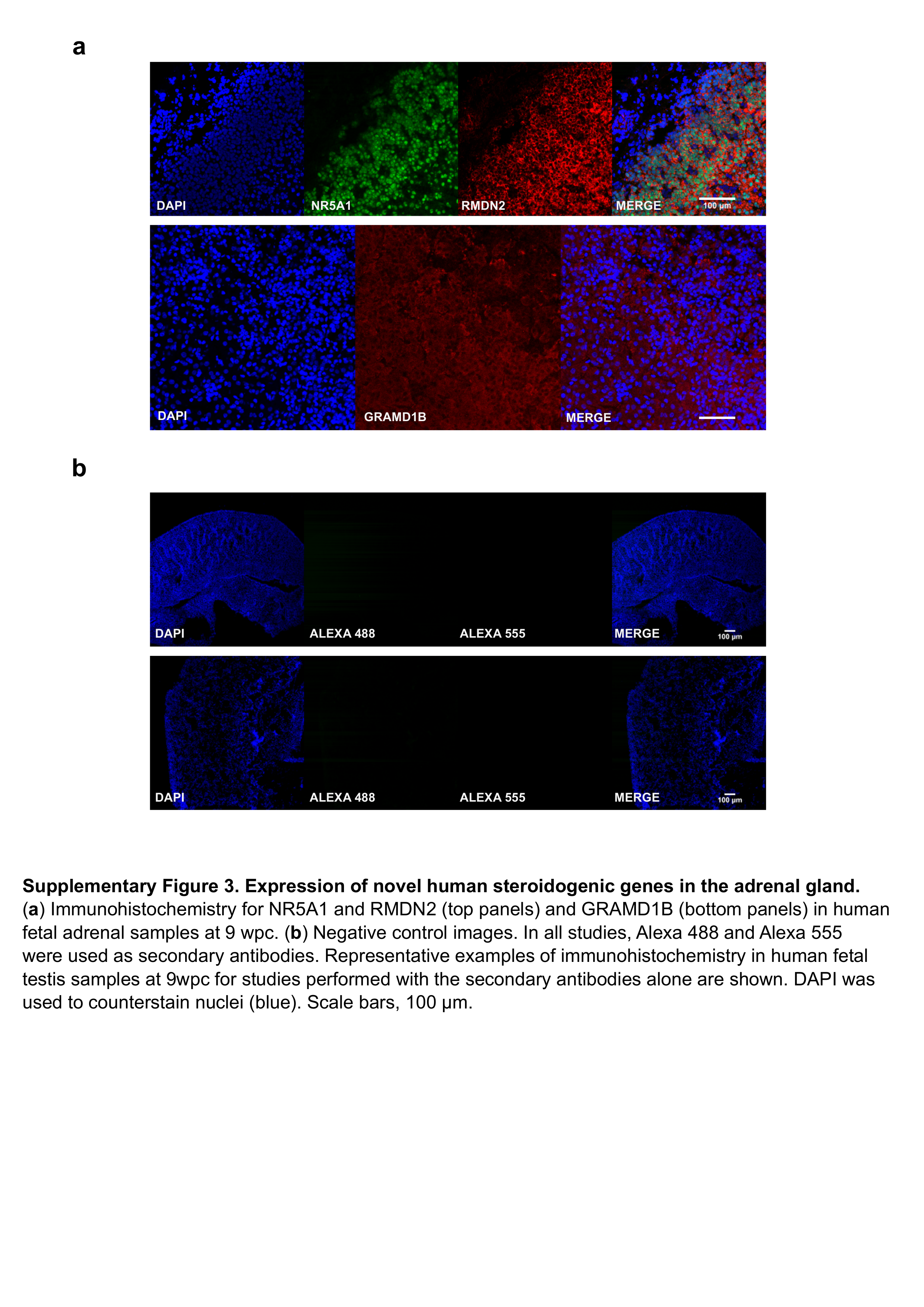

Supplement: Supplementary file 3 [file wellcomeopenres-2-14044-s0002.tgz › 296f9989-3b3a-4d10-963c-6e5a4556bf39.tiff]
