# Supplementary material for: A genomic atlas of human adrenal and gonad development
Source: Wellcome Open Res. 2017 Oct 23;2:25. Originally published 2017 Apr 7. [Version 2] doi: 10.12688/wellcomeopenres.11253.2 (PMC5407452; doi:10.12688/wellcomeopenres.11253.2)
Supplement: Supplementary file 4 [file wellcomeopenres-2-14044-s0003.tgz › b629469b-e002-4141-ac6e-ffd5645dfa05.pdf]

Supplementary Table 1. Overview of all 53 tissue samples included in the study. In this classification system, the following age ranges were used for the Carnegie Stage (CS) and Fetal (F) Stage: CS17, 41-44 dpc; CS18, 44-47.5 dpc; CS19, 47.5-50.5 dpc; CS20, 50.5-52 dpc; CS21, 52-54 dpc; CS22, 54-56 dpc; CS23, 56-58 dpc; F1 58-63 dpc (8.5-9 wpc); F2 63-70 dpc (9-10 wpc); F3, 70-77 dpc (10-11 wpc). The age used for the modelling was the midpoint of the age range for that Carnegie or Fetal Stage. Dpc: day post-conception; wpc: weeks post-conception.

Sample	Group	Batch	Carnegie Stage/Fetal Stage	Mean dpc used in modelling	Karyotype
Spine	Control	4	CS18	45.75	46,XY
Brain	Control	4	CS21	53	46,XY
Muscle	Control	4	CS23	57	46,XY
Heart	Control	4	F1	60.5	46,XY
Kidney	Control	5	F2	66.5	46,XY
Liver	Control	5	F3	73.5	46,XY
Ovary CS18	Ovary	1	CS18	45.75	46,XX
Ovary CS21	Ovary	2	CS21	53	46,XX inv(9)(p11q13)
Ovary CS23.1	Ovary	1	CS23	57	46,XX
Ovary CS23.2	Ovary	2	CS23	57	46,XX
Ovary CS23.3	Ovary	4	CS23	57	46,XX
Ovary 8.5w.1	Ovary	1	F1	60.5	46,XX
Ovary 8.5w.2	Ovary	4	F1	60.5	46,XX
Ovary 9w.1	Ovary	1	F2	66.5	46,XX
Ovary 9w.2	Ovary	2	F2	66.5	46,XX
Ovary 9w.3	Ovary	2	F2	66.5	46,XX
Testis CS18.1	Testis	1	CS18	45.75	46,XY
Testis CS18.2	Testis	1	CS18	45.75	46,XY inv(9)(p11q13)
Testis CS18.3	Testis	2	CS18	45.75	46,XY
Testis CS19	Testis	4	CS19	49	46,XY
Testis CS21.1	Testis	2	CS21	53	46,XY
Testis CS21.2	Testis	2	CS21	53	46,XY
Testis CS21.3	Testis	2	CS21	53	46,XY
Testis CS21.4	Testis	4	CS21	53	46,XY
Testis CS22	Testis	4	CS22	55	46,XY
Testis CS23.1	Testis	4	CS23	57	46,XY
Testis CS23.2	Testis	2	CS23	57	46,XY
Testis CS23.3	Testis	4	CS23	57	46,XY
Testis 8.5w.1	Testis	1	F1	60.5	46,XY
Testis 8.5w.2	Testis	4	F1	60.5	46,XY
Testis 8.5w.3	Testis	2	F1	60.5	46,XY
Testis 8.5w.4	Testis	4	F1	60.5	46,XY
Testis 9w.1	Testis	2	F2	66.5	46,XY
Testis 9w.2	Testis	2	F2	66.5	46,XY
Testis 10w.1	Testis	4	F3	73.5	46,XY
Testis 10w.2	Testis	4	F3	73.5	46,XY
Adrenal CS17	Adrenal	1	CS17	42.5	46,XY
Adrenal CS18.1	Adrenal	2	CS18	45.75	46,XY
Adrenal CS18.2	Adrenal	2	CS18	45.75	46,XY
Adrenal CS21.1	Adrenal	2	CS21	53	46,XY
Adrenal CS21.2	Adrenal	2	CS21	53	46,XY
Adrenal CS23.1	Adrenal	4	CS23	57	46,XY
Adrenal CS23.2	Adrenal	2	CS23	57	46,XY
Adrenal CS23.3	Adrenal	4	CS23	57	46,XY
Adrenal 8.5w.1	Adrenal	2	F1	60.5	46,XY
Adrenal 8.5w.2	Adrenal	2	F1	60.5	46,XY
Adrenal 8.5w.3	Adrenal	3	F1	60.5	46,XY
Adrenal 8.5w.4	Adrenal	4	F1	60.5	46,XY
Adrenal 9w.1	Adrenal	2	F2	66.5	46,XY
Adrenal 9w.2	Adrenal	2	F2	66.5	46,XY
Adrenal 9w.3	Adrenal	2	F2	66.5	46,XY
Adrenal 9w.4	Adrenal	3	F2	66.5	46,XY
Adrenal 10w	Adrenal	4	F3	73.5	46,XY
