# Supplementary material for: A genomic atlas of human adrenal and gonad development
Source: Wellcome Open Res. 2017 Oct 23;2:25. Originally published 2017 Apr 7. [Version 2] doi: 10.12688/wellcomeopenres.11253.2 (PMC5407452; doi:10.12688/wellcomeopenres.11253.2)
Supplement: Supplementary file 5 [file wellcomeopenres-2-14044-s0004.tgz › 62bd2079-19ac-47ea-8731-fe79b41c0457.pdf]

**Supplementary Table 2. Genes that are up-regulated during early testis development with a similar pattern to SOX9, ranked by amplitude of change from basal expression.**

| Gene | AIC | RSS | CNUM | B | A | L | T | B.sd | A.sd | L.sd | T.sd | RSS. NULL | F |
|---|---|---|---|---|---|---|---|---|---|---|---|---|---|
| ASPN | 108.7 | 169.6 | 50.9 | 4.32 | 2.88 | 0 | 2.05 | 0.12 | 0.15 | 0 | 0.10 | 607 | 0.28 |
| GSTA1 | 102.0 | 121.7 | 194.2 | 7.61 | 2.80 | 0 | 2.51 | 0.07 | 0.25 | 0 | 0.15 | 648 | 0.19 |
| CITED1 | 112.6 | 206.4 | 37.4 | 6.94 | 2.53 | 0 | 1.67 | 0.09 | 0.10 | 0 | 0.08 | 846 | 0.24 |
| ANKRD18A | 99.2 | 105.7 | 82.8 | 5.93 | 2.01 | 0 | 2.34 | 0.09 | 0.17 | 0 | 0.17 | 365 | 0.29 |
| MOCOS | 81.2 | 42.9 | 96.3 | 5.13 | 1.81 | 0 | 2.36 | 0.11 | 0.20 | 0 | 0.22 | 199 | 0.22 |
| HIST1H2AA | 93.3 | 78.7 | 69.9 | 4.95 | 1.79 | 0 | 2.26 | 0.10 | 0.18 | 0 | 0.20 | 237 | 0.33 |
| MAPK4 | 79.6 | 39.7 | 126.7 | 6.21 | 1.55 | 0 | 2.43 | 0.09 | 0.19 | 0 | 0.24 | 193 | 0.21 |
| NOSTRIN | 82.5 | 45.9 | 73.4 | 6.94 | 1.41 | 0 | 2.16 | 0.08 | 0.12 | 0 | 0.18 | 222 | 0.21 |
| G6PD | 92.7 | 76.3 | 55.2 | 8.79 | 1.37 | 0 | 1.98 | 0.08 | 0.12 | 0 | 0.17 | 254 | 0.30 |
| RASSF2 | 82.0 | 44.7 | 111.6 | 6.45 | 1.25 | 0 | 2.30 | 0.09 | 0.16 | 0 | 0.25 | 155 | 0.29 |
| SLC52A3 | 82.5 | 45.8 | 49.8 | 6.12 | 1.23 | 0 | 1.51 | 0.10 | 0.11 | 0 | 0.17 | 172 | 0.27 |
| KEL | 96.7 | 93.1 | 54.1 | 6.61 | 1.20 | 0 | 1.80 | 0.09 | 0.11 | 0 | 0.17 | 226 | 0.41 |
| ZNF280B | 82.0 | 44.8 | 87.4 | 7.03 | 0.98 | 0 | 1.90 | 0.08 | 0.10 | 0 | 0.21 | 142 | 0.32 |
| PRPS2 | 90.3 | 67.7 | 79.2 | 7.91 | 0.92 | 0 | 1.55 | 0.08 | 0.09 | 0 | 0.19 | 173 | 0.39 |
| **SOX9** | **86.7** | **56.5** | **94.2** | **7.45** | **0.85** | **0** | **1.67** | **0.08** | **0.09** | **0** | **0.21** | **142** | **0.40** |
| INHBB | 91.2 | 70.9 | 130.7 | 7.24 | 0.75 | 0 | 1.42 | 0.09 | 0.10 | 0 | 0.25 | 133 | 0.54 |
