# Supplementary material for: A genomic atlas of human adrenal and gonad development
Source: Wellcome Open Res. 2017 Oct 23;2:25. Originally published 2017 Apr 7. [Version 2] doi: 10.12688/wellcomeopenres.11253.2 (PMC5407452; doi:10.12688/wellcomeopenres.11253.2)
Supplement: Supplementary file 6 [file wellcomeopenres-2-14044-s0005.tgz › e5057b2a-d2bb-4056-93a4-e5e78c75e346.pdf]

Supplementary Table 3. Genes that are up-regulated around the onset of testicular steroidogenesis. B: basal value; A: amplitude of change; L: localisation (day post-conception when the maximum rate of change occurs); T: transition rate. All values shown are log2 normalised data. Genes are rated by amplitude of change (A).

Gene_name	B	A	L	T	B.sd	A.sd	L.sd	T.sd
CYP17A1	5.72	6.26	56.2	0.63	0.10	0.13	0.11	0.06
CYP11A1	7.66	3.96	57.2	0.94	0.08	0.13	0.23	0.08
LHCGR	4.08	3.83	56.8	0.25	0.10	0.12	0.13	0.15
ABCA10	4.11	3.72	60.6	1.56	0.16	0.27	0.55	0.14
INSL3	5.92	3.44	56.7	0.32	0.08	0.10	0.13	0.13
SPARCL1	6.13	3.07	56.7	1.45	0.15	0.21	0.51	0.13
CALB2	5.64	3.06	57.0	-0.10	0.08	0.09	0.10	0.27
STAR	8.17	3.06	57.7	0.69	0.07	0.12	0.25	0.11
SCARB1	7.35	3.00	56.9	0.07	0.06	0.09	0.11	0.17
ABCA8	4.27	2.96	59.7	1.21	0.12	0.17	0.46	0.13
ABCA9	4.77	2.80	57.8	1.39	0.15	0.20	0.58	0.15
C4orf49	7.08	2.72	57.6	0.79	0.07	0.12	0.27	0.11
DHCR24	9.43	2.68	59.5	0.76	0.07	0.13	0.33	0.12
HPGD	6.26	2.60	60.3	0.77	0.07	0.11	0.28	0.14
SLCO4C1	6.01	2.57	63.0	1.22	0.07	0.17	0.50	0.16
HSD17B3	5.60	2.54	54.3	0.63	0.11	0.12	0.26	0.13
PAPSS2	6.60	2.49	58.2	1.11	0.09	0.14	0.40	0.12
GPD1L	5.44	2.47	57.3	1.33	0.14	0.18	0.59	0.15
SC4MOL	8.52	2.42	59.9	0.48	0.05	0.10	0.29	0.12
FDX1	6.65	2.42	58.4	0.86	0.08	0.11	0.33	0.11
MSC	5.33	2.41	57.1	1.38	0.15	0.20	0.66	0.16
DHCR7	8.23	2.40	60.2	0.63	0.06	0.11	0.28	0.13
C11orf48	7.23	2.38	61.9	1.27	0.08	0.17	0.51	0.15
MYOCD	4.11	2.36	57.5	1.18	0.13	0.19	0.59	0.17
HSD17B6	4.15	2.35	57.6	-0.77	0.08	0.10	3.75	6.81
PRND	7.32	2.35	52.9	0.63	0.10	0.12	0.28	0.17
LDLR	8.06	2.34	58.9	0.65	0.06	0.11	0.31	0.11
IDI1	8.20	2.33	59.2	0.56	0.05	0.10	0.31	0.12
INHA	6.71	2.30	53.3	1.10	0.15	0.18	0.52	0.18
EGFLAM	6.35	2.26	56.3	0.18	0.07	0.09	0.18	0.17
GRAMD1B	7.53	2.25	57.4	0.57	0.06	0.10	0.26	0.16
ABCA6	4.15	2.22	62.1	1.40	0.13	0.26	0.89	0.25
ACSS2	7.60	2.21	60.8	0.50	0.05	0.10	0.25	0.20
LUM	5.44	2.21	57.1	0.53	0.09	0.12	0.28	0.19
MAP3K15	6.04	2.17	56.9	1.17	0.11	0.15	0.52	0.15
MVD	7.42	2.15	60.4	0.87	0.06	0.12	0.35	0.15
ADAMTS5	6.66	2.12	55.4	0.19	0.08	0.09	0.20	0.14
C7	8.30	2.11	55.4	0.24	0.07	0.09	0.20	0.14
SERPINA5	7.93	2.02	53.5	0.70	0.09	0.11	0.31	0.18
APOC1	5.66	2.00	59.1	0.65	0.08	0.11	0.40	0.15
FABP3	6.69	1.99	61.1	-1.31	0.05	0.08	-	-
HSD3B2	6.45	1.95	56.8	-0.74	0.06	0.07	0.19	1.02
FOXO4	7.79	1.95	58.1	0.90	0.07	0.12	0.41	0.14
CARTPT	4.87	1.94	56.5	-0.69	0.08	0.09	0.28	0.50
CARTPT	4.87	1.94	56.5	-0.69	0.08	0.09	0.28	0.50
EBP	7.58	1.93	61.2	1.25	0.08	0.16	0.59	0.18
VCAM1	6.49	1.93	56.6	0.48	0.08	0.10	0.27	0.20
MICA	4.81	1.93	64.1	1.40	0.10	0.28	1.18	0.30
TMEM97	8.83	1.91	57.5	1.35	0.11	0.18	0.71	0.19
DLK1	8.51	1.89	55.8	0.10	0.06	0.09	0.21	0.17
ACAT2	7.86	1.88	59.9	0.11	0.05	0.09	0.41	0.22
FLJ38894	7.66	1.88	54.1	0.81	0.09	0.12	0.36	0.18
NPC1	7.32	1.87	58.1	0.84	0.07	0.11	0.41	0.15
LSS	7.25	1.87	59.1	0.78	0.07	0.11	0.40	0.14
HMGCS1	9.46	1.85	60.2	-0.12	0.05	0.10	0.70	0.50
FDPS	7.54	1.85	60.2	0.34	0.05	0.09	0.34	0.21
PRAME	5.54	1.83	54.2	0.64	0.11	0.13	0.38	0.20
HMGCR	9.56	1.83	60.1	0.10	0.05	0.10	0.48	0.29
PLA2G16	6.80	1.83	57.1	1.48	0.14	0.20	0.83	0.20
SLC46A3	4.86	1.82	58.4	1.04	0.11	0.16	0.64	0.20
APOA1	7.91	1.82	53.6	0.51	0.08	0.10	0.27	0.19
TM7SF2	7.15	1.80	61.0	-0.02	0.05	0.09	0.53	0.94
APOA1	7.87	1.78	53.6	0.47	0.08	0.10	0.27	0.19
INSIG1	7.20	1.76	60.2	0.38	0.05	0.09	0.36	0.22
TMC5	5.97	1.75	53.8	0.48	0.10	0.11	0.33	0.20
SLC16A9	6.87	1.73	60.0	0.98	0.07	0.12	0.48	0.18
AOC3	5.25	1.72	57.0	0.17	0.09	0.11	0.25	0.36
SORCS1	5.65	1.70	55.2	-0.34	0.08	0.09	0.19	0.30
MMP9	5.34	1.67	60.1	0.65	0.08	0.12	0.49	0.23
C1S	6.93	1.65	56.8	0.99	0.09	0.12	0.51	0.18
ALAS1	8.01	1.64	56.8	0.43	0.06	0.09	0.28	0.23
LAMA2	7.63	1.62	55.5	-0.08	0.06	0.08	0.21	0.19
SCD	8.53	1.57	61.3	-1.15	0.04	0.09	-	-
CYP51A1	8.46	1.55	61.3	-1.33	0.05	0.09	-	-
SPINLW1	4.14	1.55	53.6	-0.14	0.11	0.12	0.30	0.30
SCUBE1	6.68	1.53	58.7	0.86	0.07	0.11	0.52	0.18
FRMPD1	6.01	1.53	52.8	0.66	0.12	0.13	0.52	0.28
