# Supplementary material for: A genomic atlas of human adrenal and gonad development
Source: Wellcome Open Res. 2017 Oct 23;2:25. Originally published 2017 Apr 7. [Version 2] doi: 10.12688/wellcomeopenres.11253.2 (PMC5407452; doi:10.12688/wellcomeopenres.11253.2)
Supplement: Peer review history

# Peer review: Referee response for version 2

**DOI:** 10.21956/wellcomeopenres.14044.r27229

**Published:** 2017-11-08

**Authors:** Rodolfo A. Rey, Romina P. Grinspon

**Affiliations:** 1 Centro de Investigaciones Endocrinológicas “Dr. César Bergadá” (CEDIE) CONICET – FEI – División de Endocrinología, Hospital de Niños R. Gutiérrez, Buenos Aires, Argentina; 2 Departamento de Biología Celular, Histología, Embriología y Genética, Facultad de Medicina, Universidad de Buenos Aires, Buenos Aires, Argentina

The authors have addressed all the comments satisfactorily.

We have read this submission. We believe that we have an appropriate level of expertise to confirm that it is of an acceptable scientific standard.

# Peer review: Referee response for version 2

**DOI:** 10.21956/wellcomeopenres.14044.r27230

**Published:** 2017-10-26

**Authors:** Andrew H. Sinclair, Katie Ayers

**Affiliations:** 1 Murdoch Children's Research Institute, Melbourne, VIC, Australia

We are happy to approve publication of the revised version of this manuscript.

We have read this submission. We believe that we have an appropriate level of expertise to confirm that it is of an acceptable scientific standard.

# Peer review: Referee response for version 1

**DOI:** 10.21956/wellcomeopenres.12140.r22401

**Published:** 2017-05-02

**Authors:** Jorma Toppari, Juho-Antti Mäkelä

**Affiliations:** 1 Department of Pediatrics, University of Turku, Turku, Finland; 2 Department of Physiology, University of Turku, Turku, Finland

Research manuscript entitled *“A genomic atlas of human adrenal and gonad development”* by del Valle *et al.* is an ambitious research effort and it addresses many puzzling questions concerning embryonic and early fetal gonad and adrenal gland development in human. The authors need to be thanked for taking up such a challenge and congratulated for being able to create a nicely flowing and very well written article from an enormous amount of data. The article is indeed a pleasure to read and the microarray data is presented in a clear and easily digestible manner. The article makes a substantial contribution to our canon of knowledge concerning early events in embryonic and fetal testicular, ovarian, and adrenal gland development. While it is able to corroborate earlier findings by others, it also provides an extensive amount of new data and sheds light on species-dependent differences in organogenesis. Despite these achievements, there are concerns that should be addressed in order to improve the quality of the data and make the presented arguments more scientifically sound.

 There are two major concerns that should be addressed to a satisfactory extent. Firstly, the main concern of the manuscript is data validation. Novel findings based on microarray data should always be confirmed using another method, such as qPCR and/or immunohistochemical staining (as the authors have done for the adrenal data: Fig4 and Fig 5). At many points in the manuscript (p8, p11, p25, p27) the authors propose that co-enrichment with known markers associated with distinct biological processes or monogenic disorders validates the data. This is, however, at best merely correlative data and provides only weak validation. It would greatly improve the quality of the research if the authors would validate the data at least for the most important candidate genes using qPCR and/or immunohistochemistry. The authors have included a good selection of immunohistochemical stainings but they are partially a cause for concern in their own right (see below) plus fail to provide sufficient validation for the data. The latter statement might sound harsh but in order to validate microarray data the authors need to provide at least two stainings: one that shows low/no expression at a specific time point (say CITED1 in the early testis) and high expression in another time point (CITED1 in late testis). Including this kind of data would be sufficient to address this concern and would provide the minimum acceptable validation for the microarray findings. Strictly speaking IHC does not validate microarray data as it visualizes proteins, not RNA, but at the end it is the protein expression that we are interested in and in most cases microarray/RNAseq/qPCR are just tools to identify the differentially expressed mRNAs that are translated into proteins.

 The second major concern is the quality of immunohistochemical stainings. Particularly lack of negative control stainings and difficulty to interpret the data due to inappropriate magnification and/or absence of high magnification insets. Please include negative control stainings at least for the novel markers. Provide high magnification inset for the following figures: Fig.4c top and middle panel, Fig.12c and Fig.12d. Due to poor quality, please consider providing an alternative image of higher magnification or after cropping (+inset) for Fig.12d and Fig.13d top panel. 

 Minor points:

1. P8, left column, row 12 from top. For Figure 2c FC≥2 was used, whereas for 2d FC≥1. Therefore it is a bit misleading to use term “…similar analysis…” in this case.
2. P8, right column, row 1 from top: check spelling.
3. Figure 5b. The number of samples included in the qPCR analysis is not mentioned and error bars are not shown. The authors might also consider redrawing the scale for Y axis.
4. Related to referee question #5: Is the data presented in Figure 14b and 14c available somewhere to have a look at the differentially expressed genes (cf. Figure 4b/Table 1)?
5. P26, left column, rows 5-7 from the bottom. The provided data only indicates that GRAMD1B and RMDN2 are expressed in 9 wpc testis. Because only one time point was included in the analysis, it does not demonstrate upregulation of these genes/enhanced expression of the protein products, as stated in the text.

We have read this submission. We believe that we have an appropriate level of expertise to confirm that it is of an acceptable scientific standard.

# Response

**Published:** 2017-10-17

**Authors:** John Achermann

**Affiliations:** UCL GOS Institute of Child Health, UK

**We thank the reviewers for their constructive comments. We have addressed these as shown in bold below and modified the revised manuscript accordingly.**

There are two major concerns that should be addressed to a satisfactory extent. Firstly, the main concern of the manuscript is data validation. Novel findings based on microarray data should always be confirmed using another method, such as qPCR and/or immunohistochemical staining (as the authors have done for the adrenal data: Fig4 and Fig 5). At many points in the manuscript (p8, p11, p25, p27) the authors propose that co-enrichment with known markers associated with distinct biological processes or monogenic disorders validates the data. This is, however, at best merely correlative data and provides only weak validation. It would greatly improve the quality of the research if the authors would validate the data at least for the most important candidate genes using qPCRand/or immunohistochemistry. The authors have included a good selection of immunohistochemical stainings but they are partially a cause for concern in their own right (see below) plus fail to provide sufficient validation for the data. The latter statement might sound harsh but in order to validate microarray data the authors need to provide at least two stainings: one that shows low/no expression at a specific time point (say CITED1 in the early testis) and high expression in another time point (CITED1 in late testis). Including this kind of data would be sufficient to address this concern and would provide the minimum acceptable validation for the microarray findings. Strictly speaking IHC does not validate microarray data as it visualizes proteins, not RNA, but at the end it is the protein expression that we are interested in and in most cases microarray/RNAseq/qPCR are just tools to identify the differentially expressed mRNAs that are translated into proteins.

**We agreed that further validation would be ideal at both the RNA level (qRTPCR) and protein level (immunohistochemistry), but we tried to focus on the key new genes described and were limited by the specificity of antibodies for IHC for some genes/protein of interest. We did focus largely on IHC data as we felt this provided validation at the protein level as well as RNA, but agree we could have included more validation across the time series (some of which we omitted because of space).**

**To address these issues, we have:**

**1) Provided qRTPCR data for validation of key upregulated testis genes (CITED1, ZNF280B, PRPS2, ANKRD18A) in the testis versus control samples. We also show changes in expression level across three time points (CS18, CS23, 10 wpc). These data are now shown as Supplementary Figure 1 and details have been added to the methods.**

**2) Provided qRTPCR data for upregulation of key novel steroidogenic components (GRAMD1B, FOXO4, MAP3K15, RMDN2) across time points before and after the onset of steroidogenesis (CS21, 8.5 wpc). These data are now shown as Supplementary Figure 2a and details have been added to the methods.**

**3) Unfortunately we do not have IHC data for CITED1 across time, but we have shown clear sequential upregulation by qRTPCR as described above (Supplementary Figure 1). Furthermore, we have analysed available data from single-cell transcriptomics in human gonad development recently published by Li *et al.* (Single-Cell RNA-Seq Analysis Maps Development of Human Germline Cells and Gonadal Niche Interactions. Li L, Dong J, Yan L, Yong J, Liu X, Hu Y, Fan X, Wu X, Guo H, Wang X, Zhu X, Li R, Yan J, Wei Y, Zhao Y, Wang W, Ren Y, Yuan P, Yan Z, Hu B, Guo F, Wen L, Tang F, Qiao J. Cell Stem Cell. 2017 Jun 1;20(6):858-873.e4. doi: 10.1016/j.stem.2017.03.007). We analysed all cells from 46,XY embryos/foetuses between 4 and 19 weeks gestation that cover the time course of interest (1207 single cells). Using a cut-off of >0.5 TPM to indicate expressed genes, we found that 221/1014 [21%] of SOX9 negative cells co-expressed CITED1, whereas 97/193 [50%] of SOX9 positive cells co-expressed CITED1 (Chi-squared 66.2, df =1, p-value < 4e ^-16^). Furthermore, Cited1 was identified within developing Sertoli cells in recently available data from mouse single-cell transcriptomics, with an expression time course shortly after SOX9 (Deciphering cell lineage specification during male sex determination with single-cell RNA sequencing. Stevant I, Neirijnck Y, Borel C, Escoffier J, Smith LB, Antonarakis SE, Dermitzakis ET, Nef S. http://www.biorxiv.org/content/early/2017/09/18/190264). We feel that these are additional novel, independent data to show the potential link between SOX9 and CITED1 in testis development.**

**4) We now provide sequential IHC data for GRAMD1B and RMDN2 before and after the onset of steroidogenesis to show an increase in protein expression in the cytoplasm of Leydig cells (Supplementary Figure 2b).**

**Please note that in the original manuscript we have included links to the Human Protein Atlas for many key novel factors. This resource provides IHC data for adult tissues as well as graphical data for qPCR datasets (e.g. FANTOM5, GTEx).**

The second major concern is the quality of immunohistochemical stainings. Particularly lack of negative control stainings and difficulty to interpret the data due to inappropriate magnification and/or absence of high magnification insets. Please include negative control stainings at least for the novel markers. Provide high magnification inset for the following figures: Fig.4c top and middle panel, Fig.12c and Fig.12d. Due to poor quality, please consider providing an alternative image of higher magnification or after cropping (+inset) for Fig.12d and Fig.13d top panel.

**Negative control data are particularly important to assess binding of secondary antibodies and non-specific effects. We include representative negative data in Supplementary Figure 3b. Moreover, all of the IHC images shown include areas of negative staining (e.g. capsule, non-endocrine tissue) which are important internal negative controls for the primary antibody.**

**The main aim of Fig. 4c (upper and middle panels) was to show the distribution of the factors within the adrenal in general, but not in the capsule. Higher power magnification of SF-1/NR5A1 in the adrenal gland is shown in Fig. 12c and in the new Supplementary Figure 3a, but we feel that including it as an inset would be too crowded.**

**We have included a higher power images of Fig. 12c (GRAMD1B, RMND2 in the adrenal gland) in Supplementary Figure 3a. We have added a higher magnification image of FOXO4 staining of nuclei into Figure 12d as an additional panel as requested, which shows co-expression of FOXO4 and NR5A1 in nuclei of Leydig cells. Unfortunately we are unable to provide a better quality image for 13d (upper panel) but a higher power magnification is shown.**

Minor points:

P8, left column, row 12 from top. For Figure 2c FC≥2 was used, whereas for 2d FC≥1. Therefore it is a bit misleading to use term “…similar analysis…” in this case.

**We have changed this to “analysis”.**

P8, right column, row 1 from top: check spelling.

**We are unclear what this refers to.**

Figure 5b. The number of samples included in the qPCR analysis is not mentioned and error bars are not shown. The authors might also consider redrawing the scale for Y axis.

**We agree that these data should have been presented better. We have repeated the experiment again three times in triplicate and present data with error bars. We have replaced this part of the Figure 5b.**

Related to referee question #5: Is the data presented in Figure 14b and 14c available somewhere to have a look at the differentially expressed genes (cf. Figure 4b/Table 1)?

****

**We have uploaded this dataset into OSF as Dataset 7. We apologise that this was not included.**

P26, left column, rows 5-7 from the bottom. The provided data only indicates that GRAMD1B and RMDN2 are expressed in 9 wpc testis. Because only one time point was included in the analysis, it does not demonstrate upregulation of these genes/enhanced expression of the protein products, as stated in the text.

**We have now included qRTPCR and IHC to show up-regulation over time in Supplementary Figure 2a, b.**

# Peer review: Referee response for version 1

**DOI:** 10.21956/wellcomeopenres.12140.r21686

**Published:** 2017-04-24

**Authors:** Rodolfo A. Rey, Romina P. Grinspon

**Affiliations:** 1 Centro de Investigaciones Endocrinológicas “Dr. César Bergadá” (CEDIE) CONICET – FEI – División de Endocrinología, Hospital de Niños R. Gutiérrez, Buenos Aires, Argentina; 2 Departamento de Biología Celular, Histología, Embriología y Genética, Facultad de Medicina, Universidad de Buenos Aires, Buenos Aires, Argentina

The comprehension of the mechanisms underlying adrenal and gonadal development in the first trimester of intrauterine life is essential for the understanding of human congenital disorders. Most of the information available on normal embryonic and fetal development of the adrenals and gonads derives from studies in experimental models. This study provides crucial insight into the comprehension of these developmental processes in humans. In fact, by performing microarray studies on tissues obtained from accurately staged embryos and fetuses to assess time-series changes and differential expression patterns, del Valle and colleagues have constructed an atlas of RNA expression in the adrenals, testes and ovaries from 6 to 10 weeks post-conception, a critical period in the differentiation of these organs. The authors validate their findings using quantitative RT-PCR and immunohistochemistry of genes known to be important in adrenal and gonadal development. The expression profiles of known genes were corroborated whereas unknown profiles of genes in the gonads and adrenals have been described. These findings set the bases for a wide variety of future studies addressing the physiology and pathophysiology of human adrenal and gonadal development.

 We have minor comments:

 Title and text: We have concerns about the use of the phrase “genomic atlas” in this study. In fact, genomic atlases, e.g. cancer genome atlas, refer to the comprehensive description of variants of the genome, whereas this work makes a comprehensive description of the transcripts present in the three organs at different developmental stages. The phrases “transcriptomic atlas” or “atlas of gene expression” (as used on page 5) seem more adequate.

 Methods and Results: Carnegie and fetal stages are based on the morphological development of the embryo, and are not directly dependent on either age or size. This concept should be more clearly expressed (e.g. Supplementary Table 1 and text in Methods) in order to avoid that the non-specialised reader interpret that there are precise cut-offs (e.g. CS18=44-47.5 days, etc.).

 Methods and Results: Tissue transcriptome analysis by microarray is a powerful technique. Nonetheless, like other techniques based on RNA extraction from whole tissue, one limitation is the confounding effect due to modifications in RNA levels in single components whose relative proportion in the tissue varies or differs. For instance, STAR was 73-fold higher in the adrenal tissue probably because most of the tissue sample was composed of steroidogenic cells, whereas a similar amount of testis sample would only have a few Leydig cells. This does not mean that one cortical adrenal cell expresses more STAR than one Leydig cell. Or, if Sertoli cells represent 80% of testicular tissue on week 6 and 50% on week 10, microarray results from whole testicular tissue will show a lower expression level of a given Sertoli cell RNA even if each Sertoli cell expresses the same amount of the given RNA. This limitation should be considered in the interpretation of the results: up/down-regulation give the idea of regulated increase/decrease of a given RNA expression in one cell rather than increase/decrease of RNA levels in whole tissue, which could be due to the relative proportion of cells expressing that given RNA. Therefore, the words up-regulation and down-regulation may be misleading, and may need to be replaced (e.g. “more/less abundant in whole testicular/ovarian/adrenal tissue”).

 Methods: We do not have expertise to assess the BALT model used to pinpoint the time when gene expression levels change. A mathematician/statistician should be consulted.

 Results: We would have expected SRY and SOX9 to appear as a testis differentially expressed genes on Figure 2. Could the authors discuss why they did not select them for this figure?

 Figure 6: A differential expression of SRY on the adrenal is unexpected. Could the authors comment on this?

 Suppl. Table 1: there are two samples (Ovary CS21 and Testis CS18.2) with the same abnormal karyotype translocation: 46,XX inv(9)(p11q13) and 46,XY inv(9)(p11q13) respectively. This a frequent finding in infertile patients. Could the authors discuss this finding?

 Discussion: page 25, “The role of SRY as the primary testis-determining gene has been known for 25 years, following transgenic mouse studies as well as the discovery of duplication of SRY in individuals with 46,XX testicular disorders of sex development (DSD)”. Do the authors mean translocation of SRY, or is it really duplication?

We have read this submission. We believe that we have an appropriate level of expertise to confirm that it is of an acceptable scientific standard.

## References

[1] del ValleIBuonocoreFDuncanALinLBarencoMParnaikRShahSHubankMGerrelliDAchermannJ: A genomic atlas of human adrenal and gonad development. *Wellcome Open Research*.2017;2: 10.12688/wellcomeopenres.11253.1 10.12688/wellcomeopenres.11253.1 28459107PMC5407452

# Response

**Published:** 2017-10-17

**Authors:** John Achermann

**Affiliations:** UCL GOS Institute of Child Health, UK

**We thank the reviewers for their positive and constructive comments. We have addressed these as shown in bold below and modified the revised manuscript accordingly.**

Minor comments:

Title and text: We have concerns about the use of the phrase “genomic atlas” in this study. In fact, genomic atlases, e.g. cancer genome atlas, refer to the comprehensive description of variants of the genome, whereas this work makes a comprehensive description of the transcripts present in the three organs at different developmental stages. The phrases “transcriptomic atlas” or “atlas of gene expression” (as used on page 5) seem more adequate.

**We agree that in the age of genome sequencing there might be confusion and that other terms might be more accurate, but this term has been used to describe developmental datasets before so we would prefer to leave the title as it is.**

Methods and Results: Carnegie and fetal stages are based on the morphological development of the embryo, and are not directly dependent on either age or size. This concept should be more clearly expressed (e.g. Supplementary Table 1 and text in Methods) in order to avoid that the non-specialised reader interpret that there are precise cut-offs (e.g. CS18=44-47.5 days, etc.).

**We have added a note in the methods to state that staging is based on morphological characteristics.**

Methods and Results: Tissue transcriptome analysis by microarray is a powerful technique. Nonetheless, like other techniques based on RNA extraction from whole tissue, one limitation is the confounding effect due to modifications in RNA levels in single components whose relative proportion in the tissue varies or differs. For instance, STAR was 73-fold higher in the adrenal tissue probably because most of the tissue sample was composed of steroidogenic cells, whereas a similar amount of testis sample would only have a few Leydig cells. This does not mean that one cortical adrenal cell expresses more STAR than one Leydig cell. Or, if Sertoli cells represent 80% of testicular tissue on week 6 and 50% on week 10, microarray results from whole testicular tissue will show a lower expression level of a given Sertoli cell RNA even if each Sertoli cell expresses the same amount of the given RNA. This limitation should be considered in the interpretation of the results: up/down-regulation give the idea of regulated increase/decrease of a given RNA expression in one cell rather than increase/decrease of RNA levels in whole tissue, which could be due to the relative proportion of cells expressing that given RNA. Therefore, the words up-regulation and down-regulation may be misleading, and may need to be replaced (e.g. “more/less abundant in whole testicular/ovarian/adrenal tissue”).

**We have approached this study on the “whole tissue” level and talk about up-regulation or down-regulation of genes in this regard. However, it is an important point to consider, so we have added a paragraph in the discussion to address some of the limitations of this work and included this point.**

Results: We would have expected SRY and SOX9 to appear as a testis differentially expressed genes on Figure 2. Could the authors discuss why they did not select them for this figure?

**We used a cut-off value of log2 FC >1 (i.e., two-fold) for this analysis and both factors were just below. As can be seen in Fig. 6b and 8a even changes within the tissue are subtle.**

Figure 6: A differential expression of SRY on the adrenal is unexpected. Could the authors comment on this?

**SRY may have some extra-gonadal expression, for example in the adrenal and brain. We mentioned the potential role of SRY in the regulation of renin in the discussion. This is an interesting area for future research.**

Suppl. Table 1: there are two samples (Ovary CS21 and Testis CS18.2) with the same abnormal karyotype translocation: 46,XX inv(9)(p11q13) and 46,XY inv(9)(p11q13) respectively. This a frequent finding in infertile patients. Could the authors discuss this finding?

**The inv(9)(p11q13) balanced translocation is quite a common finding in the general population (1.2-2.3%). Although data originally suggested this could be linked to infertility, more recent publications have shown that differences in 1800 infertile people were not statistically significantly higher (Association of pericentric inversion of chromosome 9 and infertility in romanian population. Dana M, Stoian V. Maedica (Buchar). 2012 Jan;7(1):25-9; see also Heteromorphic variants of chromosome 9. Kosyakova *et al.* Molecular Cytogenetics 2013;6:14). Several studies are also biased as this change can increase with age and individuals being investigated for fertility were often older. There were no specific outlying effects of these samples in our analysis, so we felt comfortable including them in the dataset.**

Discussion: page 25, “The role of SRY as the primary testis-determining gene has been known for 25 years, following transgenic mouse studies as well as the discovery of duplication of SRY in individuals with 46,XX testicular disorders of sex development (DSD)”. Do the authors mean translocation of SRY, or is it really duplication?

**Thank you for identifying this mistake. We have changed it to translocation**.

# Peer review: Referee response for version 1

**DOI:** 10.21956/wellcomeopenres.12140.r21671

**Published:** 2017-04-20

**Authors:** Andrew H. Sinclair, Katie Ayers

**Affiliations:** 1 Murdoch Children's Research Institute, Melbourne, VIC, Australia

The development of the adrenal glands and gonads represent two significant embryological events. The development of both tissues is closely linked as they both derive from the same region of intermediate mesoderm in early embryogenesis. Given this shared history it is not surprising that they share many processes such as steroidogenesis in common. However, until the current study most of our understanding of spatial and temporal gene expression profiles in these tissues has come from mouse studies which are not always applicable to humans. This study by Achermann and colleagues finally addresses this fundamental gap in our knowledge. The authors extracted RNA from adrenal, testis, ovary and control tissues between 6-10 weeks *post coitum* (wpc) in human embryos. The RNA was then subjected to an Affymetrix array and subsequent differential gene expression analysis and mathematical modelling to identify temporal changes across the dataset. In addition, they used pathway analysis and confirmed the cellular localization of novel genes using immunohistochemistry.

 The study confirmed the expression profiles of many known genes such as *NR5A1* and identified many potential novel genes. Interestingly, they showed that the only protein coding Y-chromosome gene detected in the developing human testis (CS18-CS22) was the *SRY* gene. Twenty-five years since the discovery of *SRY* as the sole primary testis determining factor we now have further proof in human tissue that this is the case. Novel components of the adrenal pathway have been identified as have genes that are potentially up-regulated by *SOX9* such as *CITED1*, *ZNF280B* and *PRPS2*. In addition, potential novel genes were associated with adrenal and testicular steroidogenesis (eg. *FOXO4*, *MAP3K15 *and *RMND2*). Of interest was the finding that while a similar number of differentially expressed genes were found in the testis and ovary, pathway-enrichment analysis failed to reveal any annotated pathways in the later. The study did reveal some interesting candidate ovarian genes such as *OR10G9* and *OR4D5*, some of which appear to be involved in neurotransmission. This clearly highlights our lack of understanding of early ovarian development, and suggests that additional methods may be required to find novel genetic pathways important in this tissue.

 The public availability of this data will be a great future resource for any researchers interested in human adrenal or gonadal development. In addition, this database may allow researchers to isolate novel genes involved in gonadal and adrenal disorders, many of which currently lack a defined genetic aetiology. Finally, while the authors used a highly comprehensive set of tissues, expression analysis using microarray platforms can have several drawbacks compared to technologies such as RNA-Seq. Microarrays have been shown to have poorer sensitivity for lowly-abundant RNA compared to RNA-Seq and may also miss expression data for poorly annotated genes and alternative transcripts. We therefore look forward to similar studies on human fetal adrenal and gonads using RNA-Seq and ultimately single cell fate mapping approaches as have been achieved in mouse.

We have read this submission. We believe that we have an appropriate level of expertise to confirm that it is of an acceptable scientific standard.

# Response

**Published:** 2017-10-17

**Authors:** John Achermann

**Affiliations:** UCL GOS Institute of Child Health, UK

**We thank the reviewers for their positive comments and outlining some limitations. We have addressed these as shown in bold below and modified the revised manuscript accordingly.**

 Finally, while the authors used a highly comprehensive set of tissues, expression analysis using microarray platforms can have several drawbacks compared to technologies such as RNA-Seq. Microarrays have been shown to have poorer sensitivity for lowly-abundant RNA compared to RNA-Seq and may also miss expression data for poorly annotated genes and alternative transcripts. We therefore look forward to similar studies on human fetal adrenal and gonads using RNA-Seq and ultimately single cell fate mapping approaches as have been achieved in mouse.

**These comments provide a very useful summary of potential drawbacks. We have included a** **paragraph in the discussion outlining some limitations of the study and future directions.**

# Peer review: Referee response for version 1

**DOI:** 10.21956/wellcomeopenres.12140.r21958

**Published:** 2017-04-19

**Authors:** Olle Söder

**Affiliations:** 1 Department of Women's and Children's Health, Karolinska Institutet, Stockholm, Sweden

This is well written, comprehensive and important work on the transcriptome of human embryonic and fetal adrenal and gonad development. It is unique in its human focus and comparative approach on adrenal, testis and ovary. Datasets and Supplementary material have been made generously available. This reviewer has only a few comments to be considered.

  

1. The mathematical model and part of the statistics used are complex and not easy to follow for a non-expert. It should therefore be reviewed by an expert statistician.
2. It is not entirely clear if data from XX adrenals were compared with data from XY adrenals and if there was any difference.
3. In Fig. 13 D, lower part, SCUBE1 expression seems to be localized to flat cells in the periphery of the chords. Have the authors considered that these cells may be peritubular cells (PTC) rather than Sertoli cells or gonocytes?  PTC may be identified by their expression of aSma and PDGF-Ra. 

I have read this submission. I believe that I have an appropriate level of expertise to confirm that it is of an acceptable scientific standard.

# Response

**Published:** 2017-10-17

**Authors:** John Achermann

**Affiliations:** UCL GOS Institute of Child Health, UK

**We thank the reviewers for their constructive comments. We have addressed these as shown in bold below and modified the revised manuscript accordingly.**

This reviewer has only a few comments to be considered.

The mathematical model and part of the statistics used are complex and not easy to follow for a non-expert. It should therefore be reviewed by an expert statistician.

**Most of the statistics used for comparing expression between samples and heatmaps use a standard R package. The model developed is also a fairly standard approach and similar profiles were obtained using soft and hard clustering analysis (where genes can be clustered to multiple or single clusters), but the model provided more detailed analysis over time.**

It is not entirely clear if data from XX adrenals were compared with data from XY adrenals and if there was any difference.

**The adrenal samples were intentionally chosen to be from 46,XY embryos/fetuses so that they could be compared directly with testis without the complicating effects of any sex-chromosome gene dosage. We agree that sex differences in extra gonadal tissues are an interesting area to study but are beyond the scope of this manuscript.**

In Fig. 13 D, lower part, SCUBE1 expression seems to be localized to flat cells in the periphery of the chords. Have the authors considered that these cells may be peritubular cells (PTC) rather than Sertoli cells or gonocytes?  PTC may be identified by their expression of aSma and PDGF-Ra.

**Thank you. At this stage of development the cells at the boundary of the cords are not particularly flattened like classic peritubular myoid cells. Certainly the cells containing SCUBE1 are fairly round and in some cases staining is seen throughout the cytoplasm and in some more centrally positioned cells. As SCUBE1 is potentially a secreted protein we wondered if the peripheral localisation was to do with this. However, we take the reviewers point that these could be developing PTM cells. We have not been able to address this further at present but will do so if we explore the role of SCUBE1 in the future.**
